# Using spectral flow cytometry to characterize anti-tumor immunity in mouse models of cancer

**DOI:** 10.1002/cpz1.70032

**Published:** 2024-10-01

**Authors:** Giampiero Valenzano, Shannon N. Russell, Simei Go, Eric O’Neill, Keaton I. Jones

**Affiliations:** 1 Department of Oncology, University of Oxford, Oxford, OX3 7DQ, UK; 2 Nuffield Department of Surgical Sciences, University of Oxford, Oxford, OX3 7DU, UK

**Keywords:** Cancer, mouse models, tumor microenvironment, immuno-oncology, spectral flow cytometry

## Abstract

Mouse models remain at the forefront of immuno-oncology research, providing invaluable insights into the complex interactions between the immune system and developing tumors. While several flow cytometry panels have been developed to study the immunity to cancer in mice, most of them present limitations in their capacity to address the complexity of anti-cancer immune responses. For example, many of the panels developed to date focus on a restricted number of leukocyte populations (notably, T cells or antigen-presenting cells), failing to include the multitude of other subsets that participate in anti-cancer immunity. In addition, these panels were developed using blood or splenic leukocytes. While the immune composition of the blood or spleen can provide information on systemic immune responses to cancer, it is in the tumor microenvironment (TME) that local immunity takes place. Therefore, we optimized this spectral flow cytometry panel for identifying the chief cell types that take part in immunity to cancer using immune cells from cancer tissue. We used pancreatic tumors implanted both orthotopically and subcutaneously to demonstrate the panel’s flexibility and suitability in diverse mouse models. The panel was also validated in peripheral immune districts (namely, the blood, spleen, and liver of tumor-harboring mice) to allow comparisons between local and systemic anti-tumor immunity.

Basic Protocol 1: Tumor induction – Orthotopic

Alternate Protocol 1: Tumor induction – Subcutaneous

Basic Protocol 2: Preparation of single cell suspensions from the tumor, spleen, liver, and blood of tumor-bearing mice

Alternate Protocol 2: Staining single cell suspensions from the tumor, spleen, liver, and blood of tumor-bearing mice

## Introduction

The intricate interplay between cancer and the immune system has been known for a long time and has received particular attention in last two decades, primarily due to the incorporation of two immunity-related hallmarks into the "Hallmarks of cancer” (Hanahan, 2022; Hanahan & Weinberg, 2011). This momentum in immuno-oncology research has catalyzed the development of various immunotherapy modalities, representing a pivotal advancement in cancer care. While immunotherapy has achieved remarkable success in hematological malignancies (Bachireddy et al., 2015; Kalos et al., 2011; Kantarjian Hagop et al., 2017; Kochenderfer et al., 2010; Tang et al., 2023), its efficacy in solid tumors, aside from highly immunogenic types such as melanoma (Knight et al., 2023) and lung cancer (Shields et al., 2021), has been more modest (Albelda, 2024; Sun et al., 2023). A substantial proportion of patients do not respond to treatment, and many who initially respond eventually relapse, highlighting the urgent need for a better understanding of anti-cancer immunity and the development of better immunotherapeutic strategies.

Mouse models remain indispensable in immuno-oncology research due to their ability to faithfully recapitulate the complex interactions between cancer and the immune system. They encompass patient-derived xenografts (PDXs), genetically engineered mouse models (GEMMs), and implantation models. PDXs, established by transplanting human cancer tissue into immunodeficient mice, retain crucial genomic and structural features of native tumors, facilitating studies on molecular targets, patient-specific drug sensitivity, and mechanisms of drug resistance (Liu et al., 2023). However, the immunodeficiency of recipient mice limits their utility in studying anti-tumor immune responses. GEMMs provide an opportunity to investigate the immune-cancer cell cross-talk from the earliest stages of tumorigenesis to advanced, metastatic disease, making them a pillar of immuno-oncology research (Kersten et al., 2017). Contrary to GEMMs, in which tumors develop spontaneously as a consequence of key driver mutations, implantation models of tumor development rely on the inoculation of cancer cell lines in immunocompetent mice. Implantation models offer distinct advantages over GEMMs: they offer easy-to-reproduce and highly standardized *in vivo* platforms for tumor development in an immunocompetent host and a shorter timeframe for tumor initiation, thereby facilitating studies in anti-cancer immunity and response to immunotherapies. In implantation models, tumor cells can be injected either orthotopically or subcutaneously. The advantages and disadvantages of each implantation model have been thoroughly reviewed elsewhere (Guerin et al., 2020; Ireson et al., 2019). The main differences between the two are briefly discussed below, in Basic Protocol 1 and Alternate Protocol 1, respectively.

Flow cytometry has historically been considered a cornerstone of immunological studies, as epitomized by the “scientific union” between cytometry and immunology being likened to a marriage (Cossarizza et al., 2021). This technology allows multiparametric, single-cell resolution analysis of immune cells in the circulation, the tumor microenvironment, as well as primary and secondary lymphoid organs. The recent advent of full-spectrum (or spectral) flow cytometry has further increased the potential of this tool. Unlike conventional flow cytometry, spectral flow cytometry entails the detection of fluorescent signals by multiple detectors across the entire emission spectrum, meaning that upwards of 40 fluorophores can be employed in a single panel (Park et al., 2020). The ability to detect a larger array of markers than previously possible removes the need for using multiple staining panels, thereby improving the detection of rare cell populations and reducing the amounts of reagents used and the relative costs. Additionally, spectral cytometers allow the extraction of cell autofluorescence, further augmenting signal resolution. Advancements in data analysis techniques have also transformed flow cytometry data analysis. Algorithms leveraging on dimensionality reduction and self-organizing clusters (Van Gassen et al., 2015) offer a more unbiased and comprehensive approach to cell classification compared to traditional manual gating. These methods enhance the efficiency of handling (and visualizing) large datasets, and help detect subtle differences and rare populations that manual gating might overlook, facilitating the discovery of novel cell subsets and their roles in the immune response.

Despite its strengths, flow cytometry has notable limitations compared to other techniques, particularly regarding the preservation of tissue architecture and the spatial organization of cells within tissues. Flow cytometry analyzes dissociated single-cell suspensions, which inherently leads to the loss of the three-dimensional spatial context of cells within their native tissue environment. This limitation precludes the examination of cell-to-cell interactions that is crucial to understanding complex biological processes, such as immune cell infiltration into tumors or tissue-specific immune responses. Techniques like imaging mass cytometry (T et al., 2023) and spatial transcriptomics (Hu et al., 2022; A. Ma et al., 2022) preserve tissue integrity and thus provide deeper insights into tissue-level immune dynamics. However, these techniques are more expensive than flow cytometry and their data analysis is less straightforward, limiting their widespread adoption.

With this article, we intend to provide practical guidance on the use of spectral flow cytometry for the analysis of anti-cancer immune responses in the setting of solid tumors. In particular, Basic Protocol 1 discusses the induction of pancreatic tumors orthotopically in immunocompetent mice with a wild-type genetic background. Alternate Protocol 1 should be used by researchers intending to induce subcutaneous, rather than orthotopic, tumors. Basic Protocol 2 describes how to obtain single cell suspensions from the tumors, spleens, livers, and blood of tumor-bearing mice. Lastly, Basic Protocol 3 details how to stain the single cell suspensions obtained in Basic Protocol 2 for flow cytometric analysis. In the introductory paragraph to Basic Protocol 3, we guide the readers through the process of panel design and optimization, which will be especially useful to researchers wishing to adapt the current panel to suit their specific experimental questions.

*NOTE: All protocols involving animals must be reviewed and approved by the appropriate animal ethics boards and regulatory bodies. All work must strictly adhere to the regulations for the care and use of laboratory animals in the country the work is performed in. Please contact your local veterinarian for advice on the induction and maintenance of anesthesia and analgesia*.

## Basic Protocol 1

### Basic protocol title: Tumor induction – Orthotopic

#### Introductory paragraph

In an orthotopic implantation model, a cancer cell line is injected into the tissue of origin of that cell line. Pancreatic cancer cell lines, for example, are normally injected into the tail of the pancreas. Implanting a cancer cell line orthotopically can pose significant technical challenges. For pancreatic cancer, this necessitates a laparotomy (a surgical incision into the abdominal cavity), making the procedure technically demanding and heavily reliant on the operator’s manual dexterity. Potential complications include hemorrhage, infection, and dissemination of cancer cells within the abdominal cavity. Although rare, tumor implantation may fail entirely, potentially due to factors such as insufficient cell numbers successfully implanted into the site, compromised cell quality, or a robust immune response that eliminates the injected cancer cells before tumor establishment. In addition, an orthotopically implanted tumor is often harder to monitor over time, with pancreatic tumor volume measurements requiring serial assessment through imaging techniques, such as ultrasound or magnetic resonance imaging (MRI), rather than through use of calipers. Nevertheless, orthotopic implantation is the preferred model when the focus of the analysis is on the tumor microenvironment (TME), as they retain all the characteristics of tumors spontaneously originating in their native anatomical location, including the infiltration of immune populations with distinct tissue tropism, which may fail to infiltrate or be supported in the subcutaneous milieu (Guerin et al., 2020). Importantly, orthotopic tumors metastasize similarly to their native counterparts, so they can be used to investigate the immune composition and response to treatment in the setting of metastatic disease (Fernandez et al., 2023; Hiroshima et al., 2016).

In this protocol, we describe how to induce pancreatic tumors orthotopically in immunocompetent mice with a wild-type (C57BL/6J) background. The pancreatic cell line for inoculation (KPC-F) was derived from tumours from the KPC GEMM, a LSL-*Kras*^G12D/+^;LSL-*Trp53*^R172H/+^;*Pdx-1*-Cre mouse (Hingorani et al., 2005). Inoculation with 500 KPC-F cells following the described protocols should lead to palpable tumours after approximately 2 weeks.

**Figure 1:**
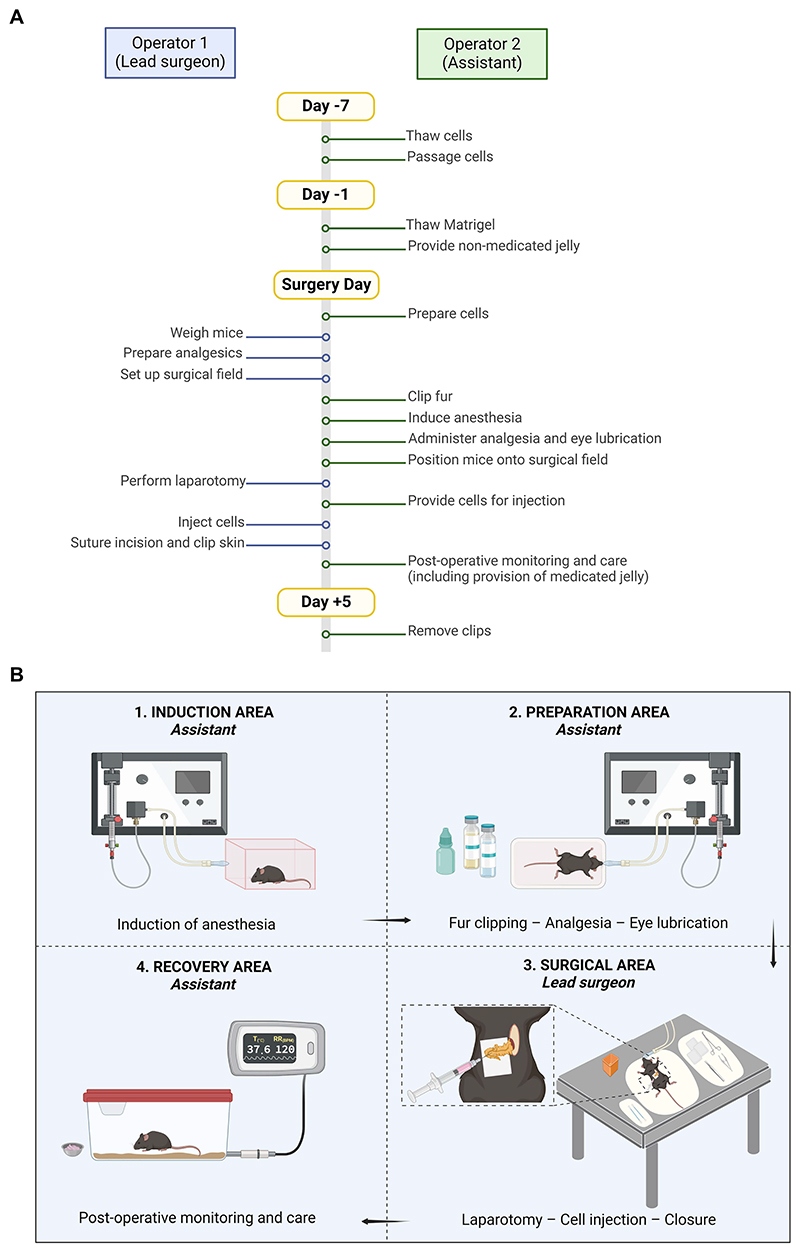
Division of duties between the lead surgeon and the surgical assistant in Basic Protocol 1. A. Timeline of events. B. Separation of spaces.

It is necessary for at least two operators to be available, a lead surgeon and surgical assistant. A recommendation for the division of tasks and the timeline for the steps in Basic Protocol 1 is presented in Figure 1. On the day of surgery, the surgical assistant prepares the cells for implantation, whilst the lead surgeon prepares the operating room and surgical area. This division of labor minimizes the amount of time cells are kept on ice prior to injection, which maintains cell quality. As the surgeon must maintain sterility once the operation has begun, the assistant will induce anesthesia, position and remove animals from the surgical field, and provide cells for injection. Having a second assistant as part of the surgical team will facilitate pre- and post-operative care but is not necessary.

#### Materials

KPC-F cells

Culture medium (see recipe in Reagents and Solutions)

Matrigel Basement Membrane Matrix (Corning, cat. no. 356231)

Wet ice

Ethanol 70% v/v

Dulbecco A Phosphate Buffered Saline, DPBS (Gibco, cat. no. 14190144)

Trypan blue (ThermoFisher Scientific, cat. no. 15250061)

Isoflurane (IsoFlo 100% w/w Inhalation Vapour, liquid, Zoetis, cat. no. 30135687, or equivalent)

Female C57BL/6J mice 7-8 weeks of age

Meloxicam (Metacam 2 mg/ml, Boheringer Ingelheim, cat. no. 30324941, or equivalent)

Buprenorphine (Vetergesic 0.3 mg/ml, Ceva, cat. no. 01300258, or equivalent)

Skin disinfectant applicator (BD ChloraPrep, cat. no. 930400, or equivalent)

Sterile ophthalmic lubricant ointment (Duratears, Bausch+Lomb, cat. no. 44101T296, or equivalent)

Medicated jelly (see recipe in Reagents and Solutions)

Non-medicated jelly (see recipe in Reagents and Solutions)

CO_2_ incubator for cell culture

Polystyrene box

Laminar flow cabinet

1.5-ml microcentrifuge tube (Eppendorf, cat. no. EP0030125150)

Hemocytometer

Light microscope

Centrifuge

Anesthetic machines

Precision balance

Heat pads

Sterile disposable surgical drapes

Autoclave (Prestige Medical 2100 or equivalent)

Autoclaved surgical tools (1 set per mouse, including 1 needle holder, 1 pair of curved, blunt scissors, 1 pair of Debakey forceps, and 1 pair of Adson forceps)

Autoclaved autoclip applier

Autoclaved, long, cotton-tipped swab

Autoclaved gauzes

Ethicon 4/0 Coated sutures (Vycril, cat. no. VCP310, 1 per 6 mice)

0.5-ml insulin syringes (BD Micro-Fine, cat. no. 324892, 1/mouse)

Sterile surgical gloves

Sharps laboratory waste container

Clipper

1-ml reduced-dead-space syringe (Braun, cat. no. 10303002)

25G needles (1/mouse)

Clip remover

#### Protocol steps with *step annotations:*

**Cell preparation in tissue culture flow cabinet (surgical assistant)**

1. Thaw one vial of KPC-F at least one week before surgery, and maintain cells in culture medium under 5% CO_2_ at 37°C. Passage cells as needed, ensuring they reach approximately 80% confluency by the day of surgery.
  *Over or under confluent cells may reduce efficacy of tumor engraftment*.
2. The day before surgery, thaw Matrigel on ice overnight.
  *It is imperative that Matrigel be thawed on ice (or at 4°C) because thawing at room temperature can compromise its integrity and functionality*.
3. On the day of surgery, wipe the polystyrene box containing Matrigel and ice with ethanol, and set it inside in the hood.
4. Transfer a pipette tip into a clean 1.5-ml microcentrifuge tube. Transfer an appropriate volume of DPBS to a sterile 1.5 mL Eppendorf tube. Place both on ice and allow to chill.
5. Lift the adherent KPC-F cells from the flask following basic cell culture protocols and perform a manual cell count: mix 10 μl of cells with 10 μl of trypan blue, load onto a hemocytometer, and count using a light microscope.
6. Centrifuge cells at 350 x *g* for 5 min, discard the supernatant, and resuspend at 5x10^5^/ml in culture medium.
7. Using the pre-chilled pipette tip transfer Matrigel into a clean 1.5-ml microcentrifuge tube, before adding the pre-chilled DPBS. Pipette up and down gently and return to ice.
  *While handling Matrigel, avoid touching the sides of the vial, as Matrigel will start to gel above 4°C. Pre-chilling pipette tips further helps maintain Matrigel in its liquid state, for ease of manipulation*.
8. Add the resuspended KPC-F cells and mix gently.
  *For 50 mice to be injected with 20 µl: 500 µl Matrigel to 450 µl DPBS to 50 µl of KPC-F cell suspension*.
9. Keep cells on ice until completion of surgery.

**Operating room and surgical area preparation (lead surgeon)**

10. Ensure the vaporiser of all anesthetic machines is filled with isoflurane and top up if necessary.
11. Weigh the mice.
12. Prepare the analgesics at a suitable concentration for pre-operative analgesia (for example, meloxicam at 5 mg/kg together with buprenorphine at 0.1 mg/kg, or variations as per local guidelines) and set aside.
13. Switch on the heat pads for the anesthesia induction chamber, the preparation area, the surgical area, and the recovery area. Set the temperature to 37°C.
14. Prepare the surgical field by placing the sterile drapes, surgical tools, autoclip applier, cotton-tipped swabs, skin disinfectant applicators, sutures, gauzes, and insulin syringes on the operating table.
  *At this stage, the surgeon must handle only the exterior of the packages containing the tools and equipment. Once a package is opened, the contents should be allowed to drop onto the surgical table. Only immediately prior to the start of surgery, after scrubbing in and donning personal protective equipment including sterile gloves, may the surgeon arrange the surgical tools in an orderly manner for the procedure*.
15. Wipe a sharps bin with ethanol and place it to the side of the surgical field, as far as possible from the sterile instruments.

**Pre-operative care (surgical assistant)**

16. Clip the fur from the upper left abdomen of the mice.
17. Turn on the anesthetic machines of the induction, preparation, and surgery areas.
18. Induce anesthesia in the induction chamber.

*Induction of anethesia can be achieved by 5% isoflurane, with 2-3% recommended for maintenance*.

19. Move the mouse to the preparation area, maintaining anesthesia with a nose cone, and administer pre-operative analgesia subcutaneously using one 1-ml syringe and 25G needle per mouse.
20. Apply the eye lubrication ointment to prevent corneal drying.
21. Place the mouse onto the surgical field in a supine position and verify the depth of anesthesia by checking for the loss of the animal's pedal withdrawal reflex prior to the start of surgery.

**Surgery (lead surgeon)**

22. Prepare the surgical area with the skin disinfectant applicator.
23. Cut out a small surgical window off a sterile drape and position it over the animal.
24. Make a single vertical incision (less than 1 cm) in the skin of the upper left abdomen.
25. Separate the skin around the incision site from the underlying abdominal wall by blunt dissection.
26. Make a second vertical incision (approximately 0.5 cm) through the muscular and peritoneal layers of the abdominal wall.
27. Exteriorize the pancreas with Debakey forceps and lay it flat onto a gauze.
  *Perform this step gently, avoid pulling or pinching the tissue too hard to prevent damage. Due to the close anatomical association between the pancreas and the spleen, the spleen will typically follow the pancreas upon exteriorization. This is a normal occurrence. Do not attempt to reposition the spleen back into the abdominal cavity or separate the two organs*.
28. Take up 20 µL of the prepared cell suspension using an insulin syringe, paying attention not to touch the vial.
  *The assistant should open the vial for the lead surgeon, after flicking it a few times to ensure cells are homogenously suspended in Matrigel*.
29. Inject the cells into the tail of the pancreas.
  *A bleb should be clearly visible at the site of the injection, confirming successful injection of cells into the pancreas. If the cells are erroneously injected onto (rather than into) the pancreas, no bleb will form*.
30. Pause for 30 seconds to allow Matrigel to solidify.
31. Gently push the pancreas (and spleen) back into the abdominal cavity.
32. Suture the muscular layer with interrupted sutures.
33. Close the skin incision with a clip applicator (1 clip for smaller incisions, 2 clips for larger incisions).

**Post-operative care (surgical assistant)**

34. Remove the mouse from the surgical area and place it in the recovery area.
35. Monitor closely until resumption of normal activity.
  *It is good practice to provide medicated jelly after surgery. Researchers wishing to adhere to this practice should initially provide non-medicated jelly on the day prior to surgery, so that mice can acclimatise to this novel foodstuff*.
36. Remove clips on day 5 post-surgery.
37. Proceed to Basic Protocol 2 upon reaching the predetermined study endpoint (or upon reaching a tumor volume of adequate size for flow cytometry analysis).

## Alternate Protocol 1

### Alternate protocol title: Tumor induction – Subcutaneous

#### Introductory paragraph

In a subcutaneous implantation model, a syngeneic cancer cell line is injected into the flank of the animal. Subcutaneous tumors less faithfully recapitulate human disease, particularly with respect to their microenvironment composition and their limited propensity to metastasize to distant tissues. However, they offer several advantages over orthotopic implantation models and are, therefore, widely used. Subcutaneous tumors are easier to implant, requiring only a simple injection in the flank and thus eliminating the need for surgery. Furthermore, the growth of subcutaneous tumors over time can easily be monitored without imaging, using calipers.

In this protocol, we describe how to induce pancreatic tumors subcutaneously in immunocompetent mice with a wild-type (C57BL/6J) background. The pancreatic cell line for inoculation is the KPC-F cell line described in Basic Protocol 1. For subcutaneous injections with KPC-F, we normally use 50,000 cells and an injection volume of 100 µL. With this dose, researchers should expect a time to tumor engraftment of approximately 2 weeks.

The division of tasks between the main operator and a second operator is less important in this protocol, and one single operator could in theory perform all the steps. Nevertheless, the presence of a second operator is beneficial.

#### Protocol steps with *step annotations*

**Cell preparation**

1. Refer to Basic Protocol 1 and adapt step 5 to obtain a concentration of 50,000 KPC-F for a final injection volume of 100 µl.
  *For 10 mice: 500 µl Matrigel, 450 µl ice-cold DPBS and 50 µl KPC-F resuspended at 1x10*^*7*^*/ml in culture medium*.

**Procedure room preparation**

2. Ensure the vaporiser of all anesthetic machines is filled with isoflurane and top up if necessary.
3. Switch on the heat pads for the anesthesia induction chamber and the injection area.
4. Wipe a sharps bin with ethanol and place it to the side of the injection area.
5. Wipe the box with the cells with ethanol and place it to the other side of the injection area.
6. Turn on the anesthetic machines of the induction and injection areas and induce anesthesia.

**Cell injection**

7. Move the mouse to the injection area, maintaining anesthesia with a nose cone.
  *For subcutaneous injections, the animal can be kept in a prone position*.
8. Take up 100 µl of the prepared cell suspension using an insulin syringe.
9. Lift the skin over the flank using Adson forceps and carefully pierce it to position the needle in the subcutaneous space.
10. Inject the cells.
  *While injecting, the Matrigel should naturally acquire a spherical shape. This can be facilitated by applying gentle pressure over the skin at the injection front with the tip of a finger*.
11. Proceed to Basic Protocol 2 upon reaching the predetermined study endpoint (or upon reaching a tumor volume of adequate size for flow cytometry analysis).

## Basic Protocol 2

### Basic protocol title: Preparation of single cell suspensions from the tumor, spleen, liver, and blood of tumor-bearing mice

#### Introductory paragraph

The non-invasive nature of blood sampling often leads researchers to use blood immune cell frequencies and functional states to investigate anti-cancer immunity. In fact, while the analysis of immune cells in the blood and other peripheral tissues (such as the spleen or liver) offers valuable insights into the systemic immune response to cancer, it cannot provide information on the immune responses taking place locally in the tumor microenvironment. As a result, this analysis should complement, rather than substitute, the examination of intratumoral immune cells.

The reason for distinguishing between blood and tissue immune responses is two-fold. Firstly, the vast majority of immune cells, including approximately 95% of T cells (Ganusov & De Boer, 2007; Kumar et al., 2018), reside and function within peripheral tissues rather than the blood (Sender et al., 2023). Secondly, immune cells in the blood and those residing in tissues often represent transcriptionally, phenotypically, and functionally distinct subsets (Masopust & Soerens, 2019; Mueller & Mackay, 2016; Sasson et al., 2020; Sojka et al., 2014). In the setting of cancer, immune cells undergo significant changes upon entering the TME, adopting complex states that are not observed elsewhere in the body(de Visser & Joyce, 2023). Thus, a comprehensive understanding of the immune response to cancer requires an integrated analysis of both systemic and intratumoral immune cells.

The analysis of cells from diverse anatomical regions necessitates organ-specific protocol adaptations that consider the unique cellular and non-cellular compositions of each organ. For example, densely fibrous tumors such as pancreatic tumors, where more than 70% of the tumor volume is made up of desmoplastic stroma, will require prolonged enzymatic digestion with proteases that break down acellular components of the extracellular matrix (ECM), helping to dissociate the tightly packed tissue and thereby liberating individual cells (Feig et al., 2012; Ferrara et al., 2021). Conversely, blood immune cells, already freely circulating in a liquid medium, require minimal processing. This protocol details the methodology for obtaining single-cell suspensions of tumor, liver, spleen, and blood immune cells for subsequent flow cytometry analysis.

**Figure 2:**
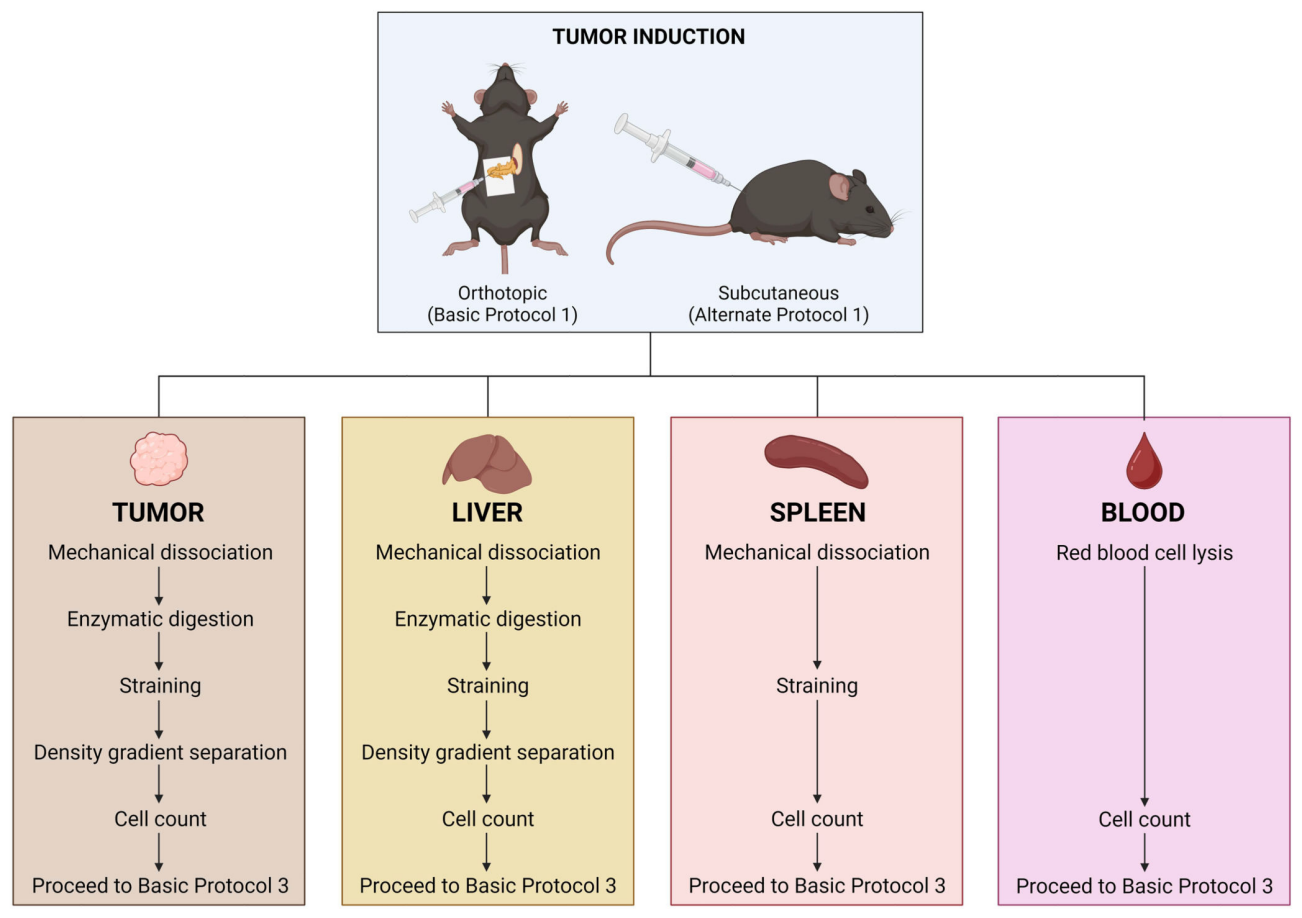
Summary of the steps involved in the processing of tumor, liver, spleen, and blood samples in Basic Protocol 2.

Figure 2 provides a schematic summary of the steps involved for each anatomical district.

*Note: The process of obtaining a cell suspension from the spleen and blood involves significantly fewer steps and less time compared to that required for the tumor or liver. For those attempting Basic Protocol 2 for the first time, we recommend adhering to the sequence of steps outlined below. With increasing familiarity (and depending on the number of samples), researchers may opt to process spleen and blood samples during the 30-minute enzymatic digestion step for tumor and liver samples. In other words, researchers may wish to perform the protocol with the following sequence of steps: 1-19 → 24-38 → 20-23*.

#### Materials

EDTA UltraPure 0.5 M (ThermoFisher Scientific, cat. no. 15575038)

Roswell Park Memorial Institute (RPMI) 1640 medium (Gibco, cat. no. 21875034)

Digestion cocktail (see recipe in Reagents and Solutions)

Ficoll-Paque PREMIUM 1.084 (hereafter referred to as “Ficoll”, Cytiva, cat. no. 17544602)

Dulbecco A Phosphate Buffered Saline, DPBS (Gibco, cat. no. 14190144)

Trypan blue (ThermoFisher Scientific, cat. no. 15250061)

Red blood cell (RBC) Lysis Buffer, 10X (BioLegend, cat. no. 420301)

7-ml Bijou containers (Starlab, cat. no. E1412)

K_2_EDTA microtainer tubes (Becton Dickinson, cat. no. 365975)

1-ml reduced-dead-space syringe (Braun, cat. no. 10303002)

27G needle

Disposable scalpel (Swann-Morton, cat. no. 0501)

Dissection tools (1 pair of curved, blunt scissors, 1 pair of sharp scissors, 1 pair of Debakey forceps, and 1 pair of Adson forceps)

Petri dishes, 35 mm, polystyrene (Sarstedt, cat. no. 83.3900.500)

15-ml tubes (Sarstedt, cat. no. 62554502)

Thermomixer Comfort (Eppendorf, or equivalent)

50-ml tubes (Sarstedt, cat. no. 62547254)

70-µm cell strainers (Falcon, cat. no. 352350)

40-µm cell strainers (Falcon, cat. no. 352340)

Centrifuge

Disposable 5-ml syringes (Braun, cat. no. 4606051V)

Serological pipettes

Pipette controller

5ml round-bottom polystyrene tubes (Falcon, cat. no. 352054)

Hemocytometer

Light microscope

96-well U-bottom plate (Sarstedt, cat. no. 821582)

Tube roller

#### Protocol steps with *step annotations:*

**Blood and tissue harvesting**

1. Label 3 Bijou containers and one K_2_EDTA microtainer tube per mouse (“Tumor”, “Liver”, “Spleen”, and “Blood” respectively).
2. Add 4 ml of plain RPMI in each Bijou container.
3. Pre-coat a 1-ml reduced-dead-space syringe with 100 µl of EDTA 0.5 M.
  Load the syringe with the 27G needle already on, so that the needle is coated, too.
4. Euthanize the animal by an approved method.
5. Collect the blood via cardiac puncture using the pre-coated syringe and needle. Transfer the blood into the labeled K_2_EDTA microtainer tube and set aside at room temperature.
6. Harvest the tumor, spleen, and liver using a scalpel and dissection tools. Transfer each organ into their respective tube and keep on ice until further processing.
  Due to the close anatomical association between the pancreas and the spleen, pancreatic tumors often abut the splenic border, particularly at larger volumes. When harvesting, take great care to separate the tumor from the spleen, meticulously cleaning the edges. The spleen is an immune-rich organ, and contamination of the tumor sample with splenocytes may confound results.

**Tumor and liver processing**

7. Transfer tumor and liver separately onto a Petri dish and mince with scissors until the tissue is finely chopped into pieces less than 1 mm in size. Continue mincing until the tissue resembles a homogenous slurry, with no discernible individual pieces.
8. Transfer the slurry into a 15 ml tube with 5 ml of digestion cocktail.
9. Incubate in the thermomixer at 37°C, with continuous shaking at 600 rpm, for 30 min (tumor) or 10 min (liver).
10. Stop enzymatic digestion by transferring the content of each 15 ml tubes into a 50-ml tube filled with 35 ml of ice-cold R10.
11. Elute the contents of the 50-ml tube through a 70-μm and a 40-μm cell strainer, sequentially.
12. Press the remaining tissue through the strainer using the back of a 5-ml syringe plunger.
13. Add 10 ml of R10 to aid elution.
14. Centrifuge at 350 x g for 5 min.
15. Label an empty 15-ml tube for each sample and add 5 ml of Ficoll.

This step can be performed during centrifugation in step 14.

16. At the end of centrifugation, discard the supernatant with a serological pipette, paying attention not to disturb the cell pellet.
17. Resuspend the cell pellet in up to 7 ml of DPBS and slowly overlay on top of the Ficoll.
  It is important to avoid mixing the cell solution in DPBS with Ficoll. We recommend setting the pipette controller to the lowest ejection speed and slowly releasing the solution along the tube walls. Begin overlaying by dispensing the cell solution drop by drop. Once a defined layer forms on top of the Ficoll, continue dispensing slowly but more freely. Tilting the tube at an angle further reduces the likelihood of mixing by creating a longer path for the liquid, which can introduce more friction and reduce the speed of downward flow.
  If a small cell pellet is obtained, reduce the volumes accordingly (e.g., 2.5 ml of DPBS and 1.5 ml of Ficoll) and use 5-ml tubes.
18. Centrifuge at 400 x g for 30 min at room temperature, with acceleration and deceleration rates both set to 0.
19. Label a 15-ml tube for each sample and add 10 ml of pre-warmed R10.

This step can be performed during centrifugation in step 18.

20. At the end of the Ficoll-based density gradient separation, collect the low-density cell fraction and transfer into the pre-labeled tubes.
  For clarity, in density gradient separation, the low-density cell fraction corresponds to the cell layer that forms between plasma and the density gradient medium (Ficoll, in this case) after centrifugation. When whole blood is separated using density gradient, this is also known as the peripheral blood mononuclear cell (PBMC) ring. On the other hand, the high-density cell fraction is the cell pellet.
21. Centrifuge at 350 x g for 5 min.
22. Discard the supernatant, resuspend in R10, and perform a manual cell count: mix 10 μl of cells with 10 μl of trypan blue, load onto a hemocytometer, and count using a light microscope.
23. Seed up to 1 million cells (at 10^7^ cells/ml) in two separate wells of a 96-well U-bottom plate and keep on ice until ready to proceed to staining (Basic Protocol 3).

The cells in one well will be stained with all the antibodies in the panel and serve as the multi-color sample, whereas the cells in the other well will not be stained and serve as the unstained, organ-specific controls.

**Spleen processing**

24. Transfer onto a Petri dish and cut with scissors until finely minced.
25. Transfer into a 15-ml tube with 10 ml of R10.
26. Elute the contents of the 15-ml tube through a 70-μm and a 40-μm cell strainer, sequentially.
27. Press the remaining tissue through the strainer using the back of a 5-ml syringe plunger.
28. Add 10 ml of R10 to aid elution.
29. Centrifuge at 350 x g for 5 min.
30. Discard the supernatant, resuspend in R10, and perform a manual cell count: mix 10 μl of cells with 10 μl of trypan blue, load onto a hemocytometer, and count using a light microscope.
31. Seed up to 1 million cells (at 10^7^ cells/ml) in two separate wells of a 96-well U-bottom plate and keep on ice until ready to proceed to staining (Basic Protocol 3).

The cells in one well will be stained with all the antibodies in the panel and serve as the multi-color sample, whereas the cells in the other well will not be stained and serve as the unstained, organ-specific control.

**Blood processing**

32. Prepare a 1X working concentration of RBC lysis buffer in deionized water.
33. Transfer the content of the K_2_EDTA microtainer tube into a 15-ml tube.
34. Rinse the microtainer tube with 200 μl of DPBS and add to the 15-ml tube.
35. Add RBC lysis buffer 1X (10 ml per ml of blood) and incubate with continuous rolling on a tube roller for 5 min.
36. Top up to 15 ml with R10 and centrifuge at 350 x g for 5 min.
37. Discard the supernatant, resuspend in R10, and perform a manual cell count: mix 10 μl of cells with 10 μl of trypan blue, load onto a hemocytometer, and count using a light microscope.
38. Seed up to 1 million cells (at 10^7^ cells/ml) in two separate wells of a 96-well U-bottom plate and keep on ice until ready to proceed to staining (Basic Protocol 3).

The cells in one well will be stained with all the antibodies in the panel and serve as the multi-color sample, whereas the cells in the other well will not be stained and serve as the unstained, organ-specific control.

## BASIC PROTOCOL 3

### Basic protocol title: Staining single cell suspensions from the tumor, spleen, liver, and blood of tumor-bearing mice

#### Introductory paragraph

Recent advancements in tumor immunology research have shed light on two crucial notions. Firstly, a broader array of cell types are implicated in tumor progression or inhibition than previously thought. Secondly, some of these cell types become resident in tissues, adopting distinct phenotypic and functional states from those of their circulating counterparts. To reflect these advances, we have designed and optimized a spectral flow cytometry panel that includes markers for the comprehensive enumeration of all key cell types involved in anti-tumor immunity, as well as markers of tissue residency.

The first step in panel design was the identification of suitable markers for the unequivocal identification of all populations and subsets of interest. This process was based on an extensive literature review. For a marker to be included in the panel, it needed to satisfy two basic requirements: its capacity to characterize—either alone or in combination—a given population of interest, and its expression in C57BL/6J mice. A list of commercially available fluorochrome-conjugated antibodies for each marker was then compiled after interrogating dedicated search engines. Each marker was assigned a tentative fluorochrome based on antigen density and fluorochrome intensity, following two general rules: 1) markers with limited commercial fluorochrome availability were assigned first, and markers with more extensive availability were assigned last; 2) markers expressed at medium or variable density were assigned to brighter fluorochromes, while markers expressed at high density were assigned to dimmer fluorochromes. A few exceptions to the second rule were made in order to ensure a more homogeneous distribution of markers across the spectrum (notably, the pairing of CD19 with APC-Fire810 and Ly6G with PE-Cy5).

Several combinations of fluorochromes were tested, and the quality of each tentative panel was assessed using two metrics: the Similarity Index (SI) and the Complexity Index (CI). The former is a measure of similarity between two fluorochrome signatures and ranges from 0 to 1; the latter is a measure of similarity across all fluorochromes in a panel and determines how efficiently different signatures can be resolved during unmixing. The closer the spectral profiles of two dyes, the more challenging it becomes to unmix them accurately, with an SI of 0.98 generally regarded as the threshold beyond which distinguishing between two fluorescent signatures becomes unreliable. The similarity and complexity indices of the definitive panel are shown in Supporting Information 1 (top pane). Another important parameter taken into consideration when pairing antigens with fluorochromes was the Stain Index Reduction (SIR) Matrix, also known as Cross-Stain Index Matrix. The SIR matrix assesses the degree of spreading error (also known as spillover) between two dyes with similar or overlapping emissions. As a general rule, combinations with higher SIR values are assigned to markers that are not co-expressed or to dump/viability channels. The SIR matrix for the definitive panel is shown in Supporting Information 1 (bottom pane), and an overview of the reagents selected for the definitive panel can be found in Table 1.

After reagents were purchased, they were individually titrated, so as to determine the optimal concentration for use. Titrations were performed on splenocytes from wild type C57BL/6J mice, except for NK1.1 Super Bright 436 and γδ TCR PerCP-eFluor710, which were titrated on the liver low-density cell fraction (largely corresponding to liver mononuclear cells) due to the low expression of these markers in the spleen. In all cases, cells were stained with the viability dye before incubation with the antibody to be titrated, so that a more accurate distinction between the positive and negative populations could be attained after gating on live singlets (as a result of the exclusion of dead cells, which bind antibodies non-specifically). The choice of the optimal concentration for use was based on a combination of visual inspection and stain index determination. A summary of the antibody titrations, including the reasoning behind the choice of optimal concentration for each reagent, is presented in Supporting Information 2.

Lastly, reference controls were obtained. Two types of controls were used for this panel: unstained and single-color. Unstained controls allow the extraction of autofluorescence and were included in every run. Tissue-specific unstained controls were recorded to reflect the slight differences in cell autofluorescence across anatomical sites (Supporting Information 3, top pane). Single-color controls, on the other hand, provide the individual signatures necessary to unmix raw data, and can be of two types: single-stained beads, and single-stained cells. The general rule for the use of single-color controls stipulates that the peak fluorescence intensity of the positive population in the control should be greater than or equal to that observed in the multi-color experimental samples. Researchers may choose to use exclusively beads, exclusively cells, or a combination of both as reference controls. The choice normally depends on both cell availability and the quality of the data generated after unmixing (i.e., the absence of unmixing errors). For our panel, we used single-stained splenocytes, due to their availability and quantity, for those markers for which the rule was satisfied. An exception was Ly6G PE-Cy5, for which we used single-stained blood leukocytes instead of splenocytes due to the greater abundance of Ly6G^+^ cells in the blood. For the remainder reagents, beads were used for unmixing.

**Figure 3:**
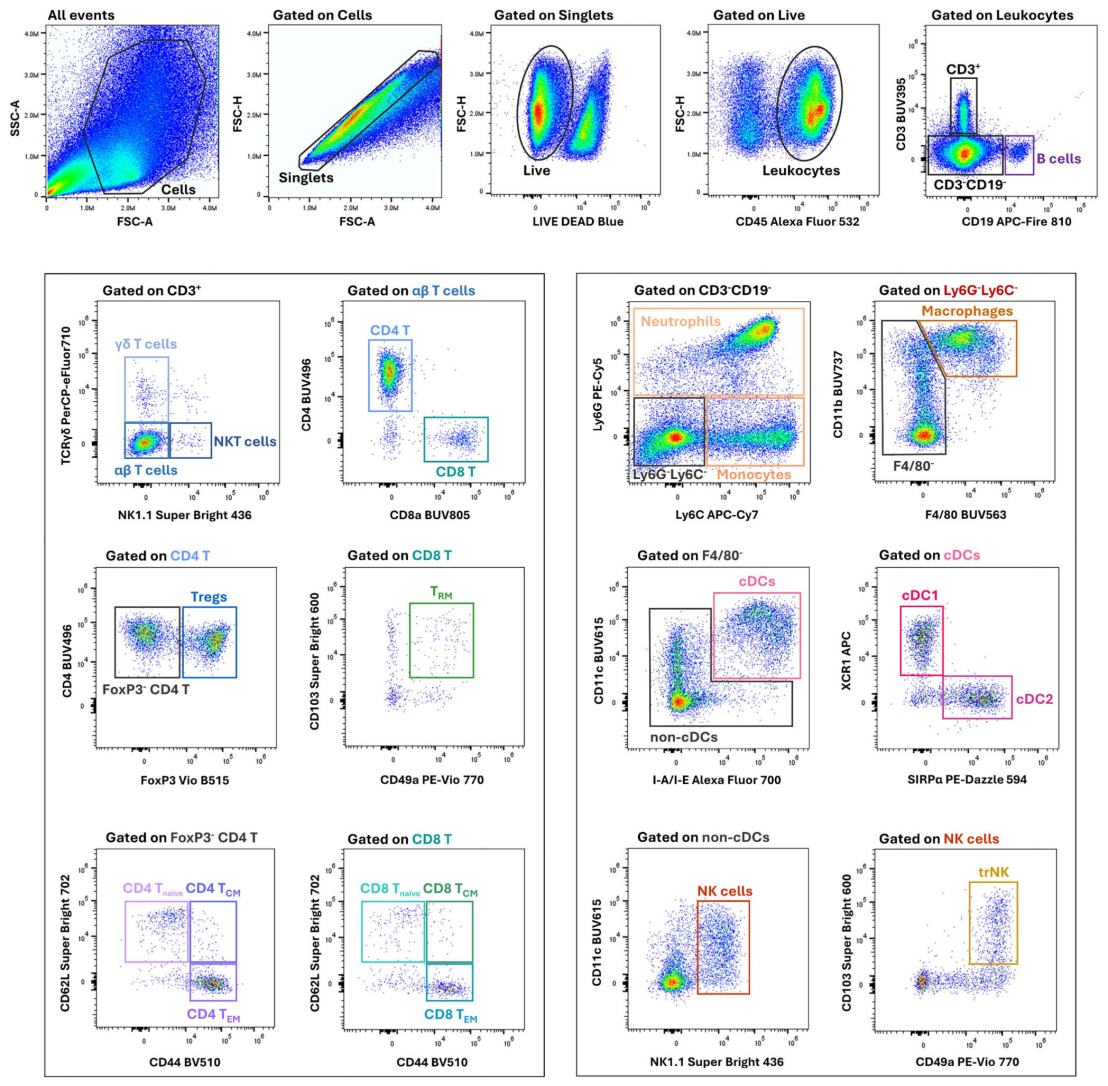
Gating strategy.

Here we provide a simple protocol for the staining of the cell suspensions obtained in Basic Protocol 2. This procedure involves the incubation of cells with a viability dye for the exclusion of dead cells, followed by incubation with antibodies targeting surface cell markers, fixation and permeabilization, and incubation with antibodies targeting intracellular markers. After staining and acquiring samples, researchers will be able to identify the key cell types involved in local and systemic anti-tumor immunity by using the gating strategy in Figure 3. A summary of the solutions to prepare during or prior to the start of the protocol can be found in Table 2. Although the volumes listed in Table 2 are specified for 1 million cells, researchers may adjust the quantities proportionally if a smaller number of cells is used.

*Note: This panel was developed using a 4-Laser (UV-V-B-R) Cytek® Aurora and data were acquired using the software SpectroFlo®. Alternative optimization may be required for other flow cytometers*.

#### Materials

Dulbecco A Phosphate Buffered Saline, DPBS (Gibco, cat. no. 14190144)

LIVE/DEAD Fixable Blue Dead Cell Stain Kit (hereafter referred to as LIVE/DEAD Blue, ThermoFisher Scientific, cat. no. L23105)

Fc Block (see Table 2)

Staining buffer (see recipe in Reagents and Solutions)

Surface antibody master mix (see Table 2)

Fixation and permeabilization agent (see Table 2)

Permeabilization buffer 10X (see Table 2)

Intracellular antibody master mix (see Table 2)

Plate centrifuge

96-well U-bottom plate (Sarstedt, cat. no. 821582)

Multi-channel pipette (200 μl)

Flow cytometer

#### Protocol steps with *step annotations*

1. Centrifuge the 96-well U-bottom plate prepared in Basic Protocol 2 at 350 x *g* for 3 min (4°C).
2. Discard the supernatant, resuspend the cell pellet in 200 μl of DPBS, and centrifuge at 350 x *g* for 3 min (4°C).
3. Discard the supernatant, resuspend the cell pellet in 100 μl of LIVE/DEAD Blue (pre-diluted with DPBS at a 1:1000 ratio), and incubate on ice for 30 min, protected from light. For unstained controls, resuspend the cell pellet in 100 μl of DPBS.

*LIVE/DEAD Blue is normally stored at -20°C. The stock vial can be thawed and diluted before the start. Alternatively, we recommend thawing the vial during centrifugation in step 1 and diluting the dye with DPBS during centrifugation in step 2, so that it is readily available by the time it is needed in step 3*.

4. Centrifuge at 350 x *g* for 3 min (4°C).
5. Discard the supernatant, resuspend the cell pellet in 250 µL of staining buffer, and repeat step 4.
6. Discard the supernatant, resuspend the cell pellet in 50 µL of Fc Block (2 µL/10^6^ cells in staining buffer), and incubate on ice for 5 min, protected from light.
7. Add 50 µL of surface antibody master mix and incubate on ice for 30 min, protected from light. For unstained controls, add 50 µL of staining buffer.

*Depending on the size of the panel, it may take some time to prepare the surface antibody master mix. We recommend preparing it before the start, or during the 30-min incubation in step 3. Once prepared, it should be stored at 4°C, protected from light*.

8. Centrifuge at 350 x *g* for 3 min (4°C).
9. Discard the supernatant, resuspend the cell pellet in 250 µL of staining buffer, and repeat step 8.
10. Discard the supernatant, resuspend the cell pellet in 200 µL of fixation and permeabilization agent, and incubate on ice for 30 min, protected from light.
11. Centrifuge at 800 x *g* for 5 min (4°C).
12. Discard the supernatant, resuspend the cell pellet in 200 µL of permeabilization buffer 1X (obtained by diluting the stock reagent with dH_2_O at a 1:10 ratio), and repeat step 11.

*We recommend preparing the 1X working concentration of the permeabilization buffer during centrifugation in step 11*.

13. Discard the supernatant, resuspend the cell pellet in 100 µL of intracellular antibody master mix, and incubate on ice for 30 min, protected from light. For unstained controls, resuspend in 100 µL of permeabilization buffer 1X.
14. Centrifuge at 800 x *g* for 5 min (4°C).
15. Discard the supernatant, resuspend the cell pellet in 400-600 µL of staining buffer, and acquire the samples on the flow cytometer.

*When acquiring samples, it is paramount to use organ-specific controls for the extraction of autofluorescence. When using SpectroFlo® for sample acquisition, it is possible to group experimental samples together (e.g*., *by anatomical district) and assign group-specific unstained controls that will be used for autofluorescence extraction*.

16. Export the .fcs files from the flow cytometer software to FlowJo (or the preferred flow cytometry analysis software), identify the major players in local and systemic anti-tumor immunity using the gating strategy in Figure 3 (see the Understanding Results section for more details).

#### Reagents and Solutions

**Culture medium**

440 ml Dulbecco's Modified Eagle Medium (DMEM, Gibco, cat. no. 21969-035)

50 ml fetal bovine serum, heat-inactivated (FBS, 10% v/v final; Sigma-Aldrich, cat. no. F9665-500ML)

5 ml L-Glutamine (2 mM final; Gibco, cat. no. 25030-024)

5 ml penicillin streptomycin (100 U/ml and 100 μg/ml final, respectively; Gibco, cat. no. 15140-122)

Mix thoroughly and filter using Millipore Steritop vacuum bottle top filter (pore size 0.22 μm, Merck, cat. no. S2GPT01RE) or equivalent.

Store up to 4 weeks at 4°C.

**Digestion cocktail**

4.85 ml Hanks' Balanced Salt Solution (HBSS, with Ca^2+^ and Mg^2+^; Gibco, cat. no. 14025092)

50 μl collagenase, type II (100 U/ml final; Gibco, cat. no. 17101015)

50 μl collagenase, type IV (100 U/ml final; Gibco, cat. no. 17104019)

50 μl DNase I, Roche (1 mg/ml final; Roche, cat. no. 10104159001)

Pre-warm HBBS to room temperature, add all enzymes, and mix thoroughly.

Prepare fresh on the day of tissue processing; do not reuse.

Amounts are for 1 tumor/liver.

**Jelly, medicated**

3.0 ml dH_2_O

3.0 g flavored gelatine

1.0 ml meloxicam (Metacam oral suspension 0.5 mg/ml; Boheringer Ingelheim, or equivalent)

Heat dH_2_O in a microwave until it reaches the boiling point, add flavoured gelatine while stirring continuously until fully dissolved, and allow to cool down.

Add buprenorphine and meloxicam to the cooled jelly solution and mix thoroughly.

Pour the medicated jelly solution into a food container, allow the solution to cool at room temperature for 10 min, and transfer the container to a refrigerator (at 4°C) for 45 min to fully solidify the jelly.

Store at 4°C for a maximum of 24h.

Amounts are for 1 food container (for a cage with 5 mice).

**Jelly, non-medicated**

3.0 ml dH_2_O

3.0 g flavored gelatine

Heat dH_2_O in a microwave until it reaches the boiling point, add flavoured gelatine while stirring continuously until fully dissolved, and allow to cool down.

Pour the medicated jelly solution into a boat-shaped container, allow the solution to cool at room temperature for 10 min, and transfer the container to a refrigerator (at 4°C) for 45 min to fully solidify the jelly.

Store at 4°C for a maximum of 24h.

Amounts are for 1 food container (for a cage with 5 mice).

**R10**

450 ml Roswell Park Memorial Institute (RPMI) 1640 medium (Gibco, cat. no. 11875093)

50 ml fetal bovine serum, heat-inactivated (FBS, 10% v/v final; Sigma-Aldrich, cat. no. F9665-500ML)

Mix thoroughly and store up to 4 weeks at 4°C.

**Staining buffer**

475 ml Dulbecco A Phosphate Buffered Saline (DPBS, Gibco, cat. no. 14190144)

25 ml fetal bovine serum, heat-inactivated (FBS, 5% v/v final; Sigma-Aldrich, cat. no. F9665-500ML)

0.5 g sodium azide (NaN_3_, 0.1% w/v final, Sigma Aldrich, cat. no. S2002)

0.2 g ethylenediaminetetraacetic acid (EDTA, 1mM final, Sigma Aldrich, cat. no. E4884)

Add EDTA and NaN_3_ to the DPBS containing 5% FBS and mix thoroughly, until completely dissolved.

Store up to 4 weeks at 4°C.

## Commentary

### Critical Parameters

#### Basic Protocol 1 and Alternate Protocol 1

*Cell line and cell preparation*

The primary factor influencing the outcome of Basic Protocol 1 and Alternate Protocol 1 is the choice of cell line for injection. This protocol has been optimized with an injection dose of 500 KPC-F in 20 μl of Matrigel (orthotopic) and 50,000 KPC-F in 100 μl of Matrigel (subcutaneous). Researchers aiming to adapt this protocol for different pancreatic cell lines or different cancer types should perform pilot studies to calibrate the injection dose and volume according to their specific experimental objectives. When testing therapeutic interventions, it is prudent to avoid very high cell doses, as they will result in the formation of excessively large and aggressive tumors that do not accurately reflect the disease's natural progression. Additionally, the cell passage number and confluency on the day of surgery are important factors. We recommend using cells that have undergone no more than 10-15 passages and have reached 80-90% confluency on the day of the procedure.

*Surgical dexterity*

The success of Basic Protocol 1 hinges on the precise execution of a surgical procedure that ensures effective tumor delivery to the implantation site while minimizing the animal’s suffering. Researchers attempting surgery for the first time should conduct this procedure under the guidance of an experienced operator and, if necessary, with the assistance of a veterinarian, so as to ensure successful tumor engraftment, maintain high standards of animal care, and reduce post-operative complications.

#### Basic Protocol 2

*Enzymatic digestion*

The reagents to be used in the enzymatic digestion step as well as its duration are entirely dependent on the composition of the organ from which a cell suspension is to be derived. This protocol was optimized for the densely fibrotic stroma of pancreatic tumors, for which two different types of collagenases and a relatively long incubation (30 min) were employed. For the digestion of the liver, on the other hand, a shorter duration (10 min) proved to be sufficient to break down ECM components and release individual cells. Researchers wishing to derive a single cell suspension from different tumors or organs than those described in this protocol should assess whether a good cell yield can be obtained using different protease combinations or shorter digestion times. It is worth noting that the same organ in a healthy and diseased state might require different digestion protocols; for example, a fibrotic/cirrhotic liver will require a longer digestion time than a healthy liver. Additionally, enzymatic digestion can partially digest surface antigens due to non-specific cleavage. Therefore, running parallel undigested controls can help verify the extent of this potential loss.

*Density gradient separation*

For optimal cell yield, it is crucial to prevent mixing between the cell solution in DPBS and Ficoll during Ficoll-based density gradient separation. We recommend strictly adhering to the instructions provided above. As a summary, we recommend:

- Setting the pipette controller to the lowest ejection speed;
- Releasing the solution along the tube walls;
- Dispensing the cell solution drop by drop at the start;
- Dispensing slowly but more freely once a defined layer forms on top of the Ficoll;
- Tilting the tube at an angle to further reduce the likelihood of mixing;
- Maintaining a ratio of cells to Ficoll greater than 1.

Adaptations of protocols for density gradient separation are found in the literature; however, these largely describe blood processing, where variations in the centrifugation speed, duration, and temperature can alter cell yield or purity (e.g., presence or absence of platelets). For solid organs, we found that spinning cells at 400G for 30 min at room temperature (with no acceleration nor deceleration) resulted in a significant enrichment for live leukocytes (discussed more in detail in Understanding results).

#### Basic Protocol 3

*Cell handling*

To maintain cell viability, it is essential to keep cells on ice, particularly when processing a large number of samples simultaneously. Pre-chilling solutions and buffers used in this protocol is crucial for maintaining a low temperature during incubation and thus preserve viability. Additionally, from the point of viability dye incubation onward, cells should be protected from light at all times to prevent photobleaching and degradation of fluorochromes.

*Antibody titration*

Antibody titration is an absolute requirement for the development of a successful flow cytometry panel. High titers can lead to increased background (due to non-specific binding) and spillover issues, whereas low titers can cause under-detection. Tables 1 and 2 provide a reference for the amounts of reagents to be used in this protocol, but any variations in reagents (including different suppliers of the same clones) should be retitrated.

*Reference controls*

Good reference controls are crucial for successful unmixing. Since this panel was designed to study the immune composition of four anatomical districts, site-specific unstained controls were used for autofluorescence extraction. As for single-color controls, we used a mix of single-stained splenocytes, white blood cells, and beads to obtain the reference fluorescent signature of all reagents in the panel. Single-stained cells shuld be used as reference controls only if the peak positive is as bright or brighter than in the multi-color experimental sample. Researchers should always verify this prior to saving a control as reference and using it for unmixing. Other key considerations when obtaining reference controls include: preventing tandem dye inter-lot variability (i.e., using the same lot in both control and experimental samples) and ensuring the autofluorescence of the positive and negative populations in a single-stain control is the same. It is common practice to use a mixture of dead and live cells as reference control for a viability dye. This practice is incorrect, as dead cells fluoresce more than live cells. As a result, for the preparation of the LIVE/DEAD Blue viability dye single-stained control, we recommend killing splenocytes (e.g., by incubation at 70°C for 45 minutes), splitting them into two halves (one stained with the viability dye and one left unstained), and then mixing them again in a single tube prior to saving as reference control.

By following the step-by-step instructions detailed above in the four protocols and paying great attention to the critical parameters, a successful outcome should be expected. However, potential issues may still arise. Table 3 summarizes the most common problems, together with their causes and solutions.

### Understanding Results

#### Tumor induction

As discussed above, the choice of the cell line for injection is a critical parameter in determining the outcome of Basic Protocol 1 and Alternate Protocol 1. Injecting 500 KPC-F in 20 μl orthotopically and 50,000 KPC-F in 100 μl subcutaneously will lead to the formation of palpable tumors approximately 14 days post-injection. When measured by ultrasound (orthotopic) or calipers (subcutaneous), tumors at this stage will have a volume of 50-100 mm^3^, with considerable variations between mice. A more detailed growth curve for orthotopically implanted tumors is provided elsewhere (Go et al., 2024). Using different cell lines will result in variations in tumor composition, time to tumor engraftment, and clinical outcomes and thus will require additional optimization prior to study commencement. Occasionally, in the orthotopic setting, a second tumor may form in the abdominal wall due to microscopic spillage during injection and heterotopic implantation. Caution should be exercised when palpating the abdomen of mice and/or measuring tumor volumes to distinguish between tumors that are correctly implanted orthotopically and the infrequent tumors that implant in the abdominal wall.

#### Preparation of single cell suspensions

**Figure 4:**
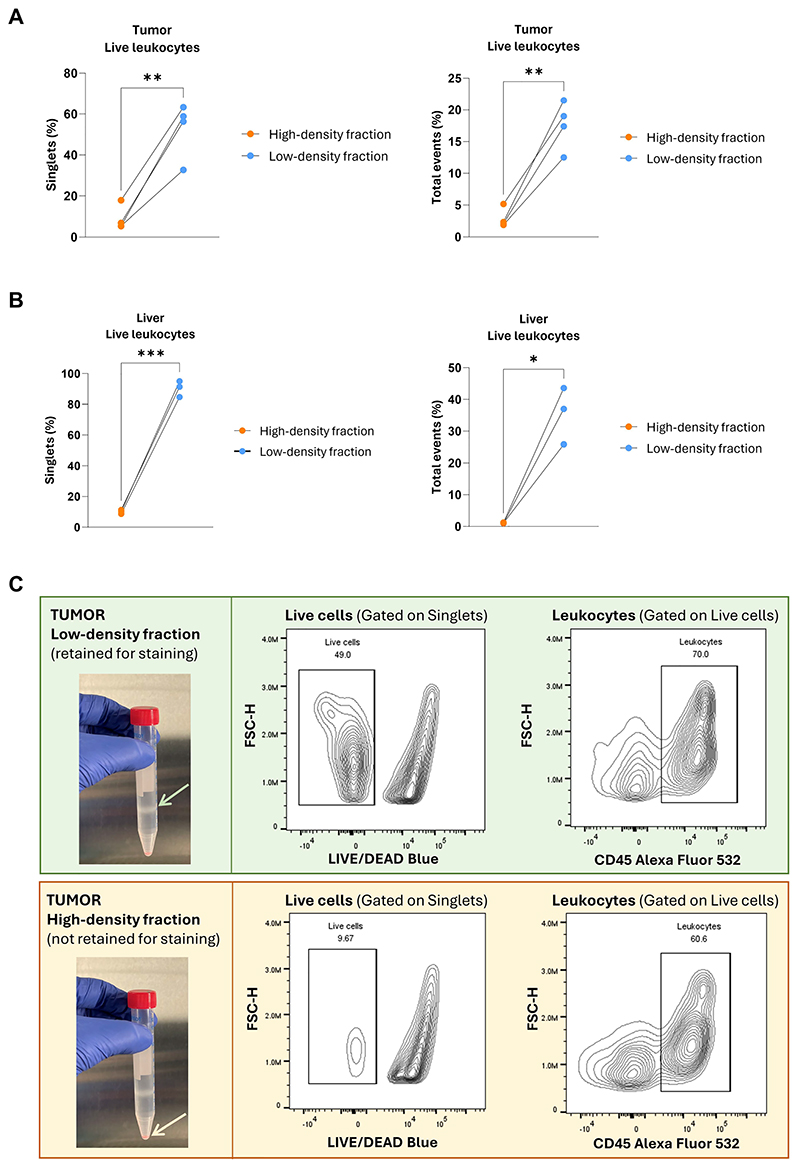
The effect of density gradient-based centrifugation on sample purity. A. Percentages of live CD45^+^ cells (live leukocytes) within single cells (left graph) and total events (right graph) in the high- and low-density cell fraction of digested tumors after density gradient-based centrifugation. Within single cells, high-density fraction: 8.9 ± 5.2 (mean ± SD); low-density fraction: 52.8 ± 11.9. Within total events, high-density fraction: 2.9 ± 1.3; low-density fraction: 17.6 ± 3.3. A paired t-test was used for all comparisons. B. Percentages of live CD45^+^ cells (live leukocytes) within single cells (left graph) and total events (right graph) in the high- and low-density cell fraction of digested livers after density gradient-based centrifugation. Within single cells, high-density fraction: 9.5 ± 0.8 (mean ± SD); low-density fraction: 89.9 ± 5.2. Within total events, high-density fraction: 1.2 ± 0.1; low-density fraction: 40.3 ± 3.3. A paired t-test was used for all comparisons. C. Representative images of a tumor sample after density-gradient centrifugation and examples of gating strategy for live cells and leukocytes in the low-density cell fraction (light green arrow, top pane) and high-density cell fraction (light orange arrow, bottom pane).

Density gradient separation is a standard procedure in the preparation of single cell suspensions when lymphocytes are the sole focus of flow cytometric analysis, as these cells are retained in the low-density cell fraction. Under physiological conditions, highly granular cells, such as neutrophils, are not retained in the low-density cell fraction but instead pellet in the high-density fraction after density gradient separation. In the setting of cancer, however, neutrophils are known to decrease their granule content and are thus retained in the low-density fraction (Bronte et al., 2016). Researchers investigating the role of neutrophils in non-cancer settings may opt to skip Ficoll separation to prevent neutrophils loss. Importantly, tumors and solid organs contain epithelial and connective tissue cells, some of which are retained after size-based straining and, therefore, stained alongside leukocytes. We found that the inclusion of a density gradient separation step led to a purer cell suspension, devoid of non-leukocyte contaminants and cellular debris, with a 6-fold enrichment in the live leukocyte fraction in tumors and a 10-to-30-fold enrichment in the live leukocyte fraction in livers (Figure 4).

The cell viability and leukocyte fraction (out of live cells) will vary across anatomical compartments. Based on our data, researchers should expect the following figures:

- A viability of 85-95%, with a leukocyte fraction of 90-100% in blood samples;
- A viability of 85-95%, with a leukocyte fraction of 80-90% in spleen samples;
- A viability of 80-90%, with a leukocyte fraction of 90-100% in liver samples;
- A viability of 50-70%, with a leukocyte fraction of 60-80% in tumor samples.

#### Identification of the populations of interest

The cell suspensions stained in Basic Protocol 3 can be analyzed using the gating strategy in Figure 3. Briefly, after the removal of cell debris and aggregates and the exclusion of dead cells using the viability dye signal, most remaining cells are leukocytes, as determined by the expression of the pan-leukocyte marker CD45. Leukocytes were classified into three major groups: B cells, CD3^+^ cells (including αβ T cells, γδ T cells, and NKT cells), and CD3^-^CD19^-^ cells (comprising all the remaining leukocyte populations).

Within CD3^+^ cells, the single expression of γδ TCR or NK1.1 defined γδ T cells and NKT cells, respectively, whereas cells in the double-negative gate (CD3^+^γδ TCR^-^NK1.1^-^) correspond to conventional αβ T cells. Two main subsets of αβ T cells were identified based on the mutually exclusive expression of the CD4 or CD8 coreceptors (CD4 T cells and CD8 T cells, respectively). Regulatory T cells (or Tregs), are then defined within the CD4 T cell subset by the expression of the forkhead box P3 (FoxP3) transcription factor. A high proportion of intratumoral Tregs, alongside a high Treg/CD8 ratio, are poor prognostic indicators in solid cancers (Iglesias-Escudero et al., 2023; Stenström et al., 2021). T cells can be subdivided into naïve (T_naïve_), central memory (T_CM_), effector memory (T_EM_), and tissue-resident memory T cells (T_RM_). The first three subsets are defined based on the expression of CD44 and CD62L (T_naïve_ being CD44^-^CD62L^+^, T_CM_ being CD44^+^CD62L^+^, and T_EM_ being CD44^+^CD62L^-^) and have been described within both the CD4 T and CD8 T cell subsets. On the other hand, the concept of tissue residency has been studied more extensively within the CD8 T cell pool, where the expression of CD49a and CD103 is used to define residency at epithelial sites (Bergsbaken & Bevan, 2015; Mackay et al., 2015). Of note, CD69 is often used as a surrogate for tissue residency, but because this marker is also upregulated transiently soon after activation on circulating (non-resident) cells, we decided against its inclusion in this panel. The tissue-resident memory subset has been associated with tumor control (Han et al., 2020; Okła et al., 2021; Yenyuwadee et al., 2022); for example, the number of tissue-resident lymphocytes was found to increase towards the centre of human lung cancers (Brownlie et al., 2023). It is important to note that the CD44/CD62L and the CD103/CD49a biaxial gates are not mutually exclusive; therefore, there will be some overlap between the subsets identified within them.

Within CD3^-^CD19^-^ cells, we defined neutrophils as Ly6G^+^ cells and monocytes as Ly6C^+^Ly6G^-^ cells. The CD11b^+^Ly6C^lo^ subset of neutrophils and the CD11b^+^Ly6C^hi^ subset of monocytes correspond phenotypically to polymorphonuclear- and monocytic-myeloid-derived suppressor cells (MDSCs), respectively. However, functional assays are necessary to confirm the designation of these cells as MDSCs, according to the latest recommendations (Bronte et al., 2016). MDSCs are key promoters of tumor progression; their accumulation within the tumor microenvironment has been associated with impaired tumor control by T and NK cells, stimulation of angiogenesis, and resistance to immunotherapy (Lasser et al., 2024). We did not include markers for the identification of other granulocytes (eosinophils, basophils, and mast cells) because of their primary role in mediating allergic reactions and innate immune responses against pathogens, rather than tumors. Readers wishing to investigate their role in anti-tumor immunity are advised to use of the following markers: Siglec-F for eosinophils, FcεRIa for basophils, and CD117 (also known as c-Kit) for mast cells.

Macrophages are defined within the CD3^-^CD19^-^Ly6G^-^Ly6C^-^ population based on the expression of CD11b and F4/80. In tumor samples, these cells can be referred to as tumor-associated macrophages (TAMs). While we have included two markers for the characterization of TAMs in the panel (CD86, primarily expressed by M1-like polarized TAMs, and Arginase-1, primarily expressed by M2-like polarized TAMs), researchers should expect a mixture of TAMs expressing either, neither, or both markers. This is because TAMs exhibit extraordinary plasticity in the TME, with the M1- and M2-polarized states representing the extremes of a broader, heterogenous spectrum (Aras & Zaidi, 2017; R.-Y. Ma et al., 2022).

Among F4/80^-^ cells, we defined conventional dendritic cells (cDCs) as cells expressing both CD11c and I-A/I-E. We further classified cDCs into type 1 cDCs (cDC1) and type 2 cDCs (cDC2) based on the mutually exclusive expression of the chemokine receptor XCR1 and the myeloid inhibitory receptor signal regulatory protein α (SIRPα), following the guidelines for dendritic cell preparation and flow cytometry analysis of mouse nonlymphoid tissues (Probst et al., 2023). Due to their capacity to cross-present tumor antigens to naïve CD8 T cells, cDC1 have been the focus of many immuno-oncology studies, and are thoroughly reviewed elsewhere (Audsley et al., 2020; Noubade et al., 2019). However, similar to macrophages, recent studies have shown that the cDC1/cDC2 dichotomy may oversimplify a more complex landscape of dendritic cell heterogeneity within tumors and other markers may emerge in the future to define the tumor-specific functional states adopted by dendritic cells *in situ* (Heras-Murillo et al., 2024). Lastly, NK cells were defined by the expression of NK1.1 and tissue-resident NK cells (trNK) were further classified using the same markers used for T cells (CD49a and CD103).

#### Data visualization and analysis

**Figure 5:**
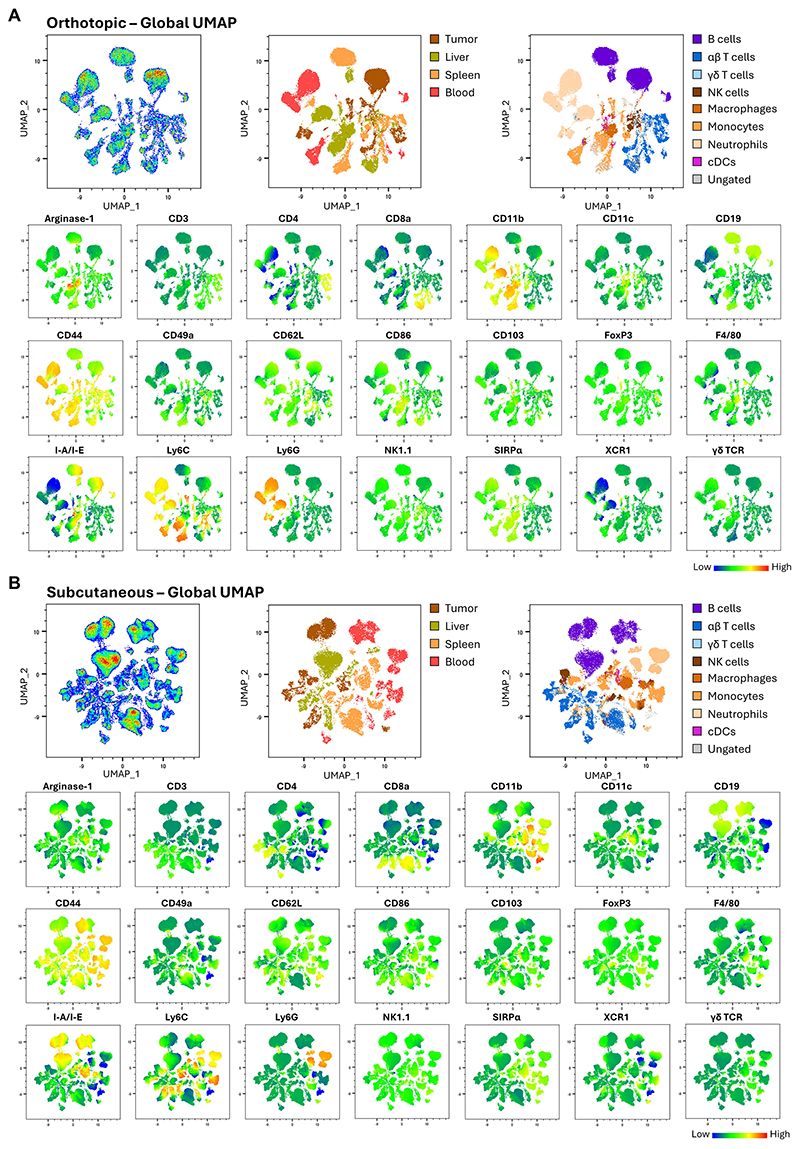
Data visualization using dimensionality reduction A. Orthotopic global Uniform Manifold Approximation and Projection (UMAP). Top pane, left: Global pseudocolor plot showing UMAP dimensionality reduction applied upon concatenating samples from the four examined anatomical districts, using the UMAP_R FlowJo Plugin (Distance metric: Euclidean; Nearest Neighbors: 15; Min. Distance: 15). Only live, CD45^+^ cells were used. All parameters except LIVE/DEAD Blue and CD45 were included in the concatenation and dimensionality reduction. Top pane, centre: Global UMAP showing the cell of origin based on anatomical district. The color coding is the one used in Figure 2. Top pane, right: Global UMAP showing the major populations identified by manual gating. Bottom pane: Global UMAP plots showing the expression of every marker in the panel (except LIVE/DEAD Blue and CD45). B. Subcutaneous global Uniform Manifold Approximation and Projection (UMAP). Top pane, left: Global pseudocolor plot showing UMAP dimensionality reduction applied upon concatenating samples from the four examined anatomical districts, using the UMAP_R FlowJo Plugin (Distance metric: Euclidean; Nearest Neighbors: 15; Min. Distance: 15). Only live, CD45^+^ cells were used. All parameters except LIVE/DEAD Blue and CD45 were included in the concatenation and dimensionality reduction. Top pane, centre: Global UMAP showing the cell of origin based on anatomical district. The color coding is the one used in Figure 2. Top pane, right: Global UMAP showing the major populations identified by manual gating. Bottom pane: Global UMAP plots showing the expression of every marker in the panel (except LIVE/DEAD Blue and CD45).

Once all populations of interest have been identified as described above, insights into local and systemic anti-tumor immunity can be derived by calculating absolute and relative cell counts, cell fractions, and levels of marker expression, using mean fluorescent intensity (MFI) as a surrogate for expression levels. In recent years, with the increasing number of markers per panel, data visualization techniques that provide a comprehensive overview of data have become more common. Figure 5 illustrates the use of dimensionality reduction to visualize data in the orthotopic (Figure 5A) and subcutaneous (Figure 5B) settings. First, samples from all four anatomical districts were concatenated into a single file. Then, Uniform Manifold Approximation and Projection (UMAP) dimensionality reduction was applied to the concatenated file (top pane, left). Various types of information can be displayed on the UMAP plot, such as the cells' origin based on the anatomical district (top pane, center), the populations identified by manual gating (top pane, right), or the expression of each individual marker in the panel (bottom pane). UMAP plots can also be used as the starting point for unbiased methods of data visualization and cell annotation (Van Gassen et al., 2015), which are becoming increasingly popular.

### Time Considerations

An overview of the steps involved in each protocol, with their duration and additional comments is presented in Table 4.

## Supplementary Material

Supporting Information 1

Supporting Information 2

Supporting Information 3

Supporting Information

## Figures and Tables

**Table 1:** Overview of reagents in the spectral flow cytometry panel.

Antibody	Clone	Purpose	Catalogue number	Supplier	Concentration (ng per 100 μL per 10^6^ cells)	Staining step
Arginase-1 PE	W21047I	Macrophage subset	165804	BioLegend	7.8	Intracellular
CD3 BUV395	17A2	T cell lineage	363-0032-82	ThermoFisher	500	Surface
CD4 BUV496	RM4-5	T cell subset	364-0042-82	ThermoFisher	100	Surface
CD8a BUV805	53-6.7	T cell subset	368-0081-82	ThermoFisher	250	Surface
CD11b BUV737	M1/70	Myeloid lineage	367-0112-82	ThermoFisher	125	Surface
CD11c BUV615	N418	Dendritic cell lineage	366-0114-82	ThermoFisher	125	Surface
CD19 APC-Fire 810	6D5	B cell lineage	115578	BioLegend	100	Surface
CD44 BV510	IM7	T cell subset	103044	BioLegend	250	Surface
CD45 AF532	30-F11	Pan-Leukocyte marker	58-0451-82	ThermoFisher	62.5	Surface
CD49a PE-Vio770	REA493	Tissue residency	130-123-892	Miltenyi Biotec	93.8	Surface
CD62L SB702	MEL-14	T cell subset	67-0621-82	ThermoFisher	25	Surface
CD86 BV785	GL-1	Macrophage subset	105043	BioLegend	62.5	Surface
CD103 SB600	2E7	Tissue residency	63-1031-82	ThermoFisher	500	Surface
FoxP3 VioB515	REA788	T cell subset	130-111-681	Miltenyi Biotec	23.4	Intracellular
F4/80 BUV563	BM8	Macrophage lineage	365-4801-82	ThermoFisher	1000	Surface
I-A/I-E AF700	M5/114.1 5.2	Dendritic cell lineage	107622	BioLegend	250	Surface
LIVE/DEAD Blue	NA	Viability	L34962	ThermoFisher	NA (use at 1:1000)	Dead cell staining
Ly6C APC-Cy7	HK1.4	Monocyte lineage	128026	BioLegend	100	Surface
Ly6G PE-Cy5	1A8	Neutrophil lineage	127672	BioLegend	250	Surface
NK1.1 SB436	PK136	NK cell lineage	62-5941-82	ThermoFisher	250	Surface
SIRPα PE-Dazzle594	P84	Dendritic cell subset	144015	BioLegend	250	Surface
XCR1 APC	REA707	Dendritic cell subset	130-111-373	Miltenyi Biotec	187.5	Surface
γδ TCR PerCP-eF710	eBioGL3	T cell subset	46-5711-82	ThermoFisher	31.3	Surface

**Table 2:** Preparation of reagents and solutions for Basic Protocol 3.

LIVE/DEAD Blue		
Reagent	Catalogue no.	μl per 10^6^ cells
LIVE/DEAD Fixable Blue Dead Cell Dye	L34962	0.1
DPBS	14190144	99.9

Fc Block		
Reagent	Catalogue no.	μl per 10^6^ cell
Mouse BD Fc Block	553141	2
Staining buffer	NA (See recipe)	48

Surface Antibody Master Mix		
Reagent	Catalogue no.	μl per 10^6^ cells
CD3 BUV395	363-0032-82	2.5
CD4 BUV496	364-0042-82	0.5
CD8a BUV805	368-0081-82	1.25
CD11bBUV737	367-0112-82	0.625
CD11c BUV615	366-0114-82	0.625
CD19 APC-Fire810	115578	0.5
CD44 BV510	103044	1.25
CD45 AF532	58-0451-82	0.313
CD49a PE-Vio770	130-123-892	0.625
CD62LSB702	67-0621-82	0.125
CD86 BV785	105043	0.313
CD103SB600	63-1031-82	2.5
F4/80 BUV563	365-4801-82	5
I-A/I-E AF700	107622	0.5
Ly6C APC-Cy7	128026	0.5
Ly6G PE-Cy5	127672	1.25
NK1.1 SB436	62-5941-82	1.25
SIRPα PE-Dazzle594	144015	1.25
XCR1 APC	130-111-373	1.25
γδ TCR PerCP-eF710	46-5711-82	0.157
Super Bright Staining Buffer	SB-4401-42	10
Staining buffer	NA (See recipe)	17.7

Fixation and permeabilization agent		
Reagent	Catalogue no.	μl per 10^6^ cells
Fixation/Permeabilization concentrate	00-5123	50
Fixation/Permeabilization diluent	00-5223	150

Permeabilization buffer 1X		
Reagent	Catalogue no.	μl per 106 cells
Permeabilization buffer 10X	00-8333-56	20
dH_2_O	NA	180

Intracellular Antibody Master Mix		
Reagent	Catalogue no.	μl per 106 cells
Arginase-1 PE	165804	0.04
FoxP3 VioB515	130-111-681	0.156
Permeabilization buffer 1X	See above	99.8

**Table 3:** Troubleshooting.

Problem	Possible Cause	Solution
Basic Protocol 1: Cells are erroneously injected onto (rather than into) the pancreas. This will lead to cancer cell dissemination into the abdominal cavity.	Needle positioned incorrectly (piercing through the tissue instead of remaining within it).	The entire organ should be immediately flushed with sterile water using a 1-ml syringe equipped with a wide bore (22G to 24G) needle. This will result in death of the cancer cells by osmotic shock. A second injection can be attempted after the flush.
Basic Protocol 1: Bleeding	Damage to local blood vessels.	Use a cotton swab to apply gentle pressure on the source of bleeding. Upon achievement of hemostasis, the procedure can continue.
Basic Protocol 2: The contents of the 50-ml tube do not elute through the strainer.	The solution is highly cellular (high cell density in the digested tissue or large size of the tumor/liver).	Use a 1,000-μl pipette tip to stir the solution, and move the larger, undigested bits of tissue towards the side of the strainer. If necessary, further dilute the solution by adding more R10.
Basic Protocol 2: The cell suspension from the digested liver and/or tumor and Ficoll admix during centrifugation.	The acceleration and/or deceleration rates were not set to 0, disrupting the formation of layers.	Immediately stop the centrifuge run, mix the sample by gently inverting the tube a few times and layer over Ficoll again, ensuring the acceleration and deceleration rates are both set to 0.
Basic Protocol 3: The sample viability observed on the flow cytometry analysis software is considerably higher than that recorded during manual cell count in Basic Protocol 2.	Cells were incubated with the viability dye in a protein-rich medium, causing the amine-reactive viability dye to bind non-specifically to the proteins in the medium and reducing the amount of dye available to bind to dead cells.	Repeat staining using DPBS in the viability dye incubation step.
Basic Protocol 3: The sample viability observed on the flow cytometry analysis software is considerably lower than that recorded during manual cell count in Basic Protocol 2.	Cell loss during handling and staining or excessive fixation.	Repeat staining ensuring: Adherence to the manufacturer’s instructions for the use of the viability dye and fixation/permeabilization reagent. Titration of the viability dye. Appropriate cell handling (e.g., keeping cells on ice).
Basic Protocol 3: Unmixing errors are observed (e.g., hypernegative populations or oddly-shaped negative populations).	Reference controls of inadequate quality were used during unmixing.	Repeat unmixing with autofluorescence extraction, ensuring: The unstained controls are from the same anatomical district as the multi-color experimental samples. The mean fluorescent intensity (MFI) of the positive population in the single-stained controls is greater than or equal to that of the positive population in the multi-color experimental samples. All controls are treated in exactly the same way as experimental samples (e.g., same duration of fixation and incubation with antibodies).

**Table 4:** Time considerations.

Basic protocol 1		
Protocol step	Duration	Additional notes
Thaw and passage cells	15 min / passage	Start at least 1 week before surgery; account for extra time when preparing sterile culture medium (30 min).
Thaw Matrigel	Overnight	1 day before surgery.
Prepare and provide non-medicated jelly (optional)	60 min	1 day before surgery; account for cool-down time (10 min) and refrigerator time (45 min).
Count and prepare cells for injection	30 min	Day of surgery, to be done by assistant while surgeon prepares the room.
Room and surgical field preparation	30 min	Day of surgery, to be done by surgeon while assistant prepares cells for injection.
Pre-operative care (mice)	30 min	Day of surgery, to be done by assistant while lead surgeon transports cells to animal facility.
Surgery	10 min / mouse	Account for more time in case of complications, or less time as familiarity with the procedure increases.
Prepare and provide medicated jelly (optional)	60 min	Day of surgery; account for cool-down time (10 min) and refrigerator time (45 min).
Clip removal	10 min	Day 5 post-surgery.

Alternate protocol 1		
Protocol step	Duration	Additional notes
Thaw and passage cells	15 min / passage	Start 1-2 weeks before surgery; account for extra time when preparing sterile culture medium (30 min).
Thaw Matrigel	Overnight	1 day before surgery.
Count and prepare cells for injection	30 min	Day of surgery, to be done by lead surgeon while assistant prepares room.
Room preparation	10 min	Day of surgery, to be done by assistant while lead surgeon prepares cells for injection.
Cell injection	5 min / mouse	Account for less time as familiarity with the procedure increases.

Basic protocol 2		
Protocol step	Duration	Additional notes
Prepare buffers	30 min	Thaw collagenase II, collagenase IV, DNase, and FBS; bring HBBS to room temperature. Prepare digestion cocktail and R10.
Label and coat tubes	5 min	
Euthanize mice and collect blood	3-5 min / mouse	Keep at room temperature until further processing.
Harvest tumor and organs	3-5 min / mouse	Keep on ice until further processing.
Tumor and liver: mechanical dissociation	3-5 min / organ	
Tumor: enzymatic digestion	30 min	Bring the thermomixer to 37°C prior to start, incubate alongside liver samples.
Liver: enzymatic digestion	10 min	Bring the thermomixer to 37°C prior to start, incubate alongside tumor samples.
Tumor and liver: straining	20 min	Including sequential elution and a 5-min centrifugation step.
Tumor and liver: density gradient separation	45 min	Including time to slowly overlay cells onto Ficoll (5 min / sample).
Tumor and liver: seed low-density cell fraction in staining plate.	15 min	Including a 5-min centrifugation step and cell count.
Spleen: mechanical dissociation	3-5 min / organ	This and the following steps can be performed during tumor and liver density gradient separation as familiarity with the procedure increases.
Spleen: straining	20 min	Including sequential elution and a 5-min centrifugation step.
Spleen: seed cells in staining plate.	15 min	Including a 5-min centrifugation step and cell count.
Blood: RBC lysis	10 min	Including preparation of 1X working concentration of RBC lysis buffer. This can be performed concomitantly with spleen processing as familiarity with the procedure increases.
Blood: seed cells in staining plate.	15 min	Including a 5-min centrifugation step and cell count

Basic protocol 3		
Protocol step	Duration	Additional notes
Prepare reagents	Variable (approx. 30 min)	Mandatory: thaw and dilute LIVE/DEAD Blue and prepare staining buffer. Optional: Prepare Fc block, fixation and permeabilization agent, permeabilization buffer 1X, as well as surface and intracellular antibody master mixes. These reagents can be prepared either prior to the start or during later incubation steps. Store at 4°C, protected from light if appropriate.
Incubate cells with viability dye	40 min	Including washes.
Incubate cells with Fc block	5 min	
Incubate cells with surface antibody master mix	40 min	Including washes.
Incubate cells with fixation and permeabilization agent	40 min	Including washes.
Incubate cells with intracellular antibody master mix	40 min	Including washes.
Acquire samples, export .fcs files and analyze	Variable	

## Data Availability

Data are available from the corresponding author upon reasonable request.
