# Supplementary figures and images for: Using spectral flow cytometry to characterize anti-tumor immunity in mouse models of cancer
Source: Curr Protoc. Author manuscript; Available in PMC 2025 Aug 12. (PMC7617995; doi:10.1002/cpz1.70032)

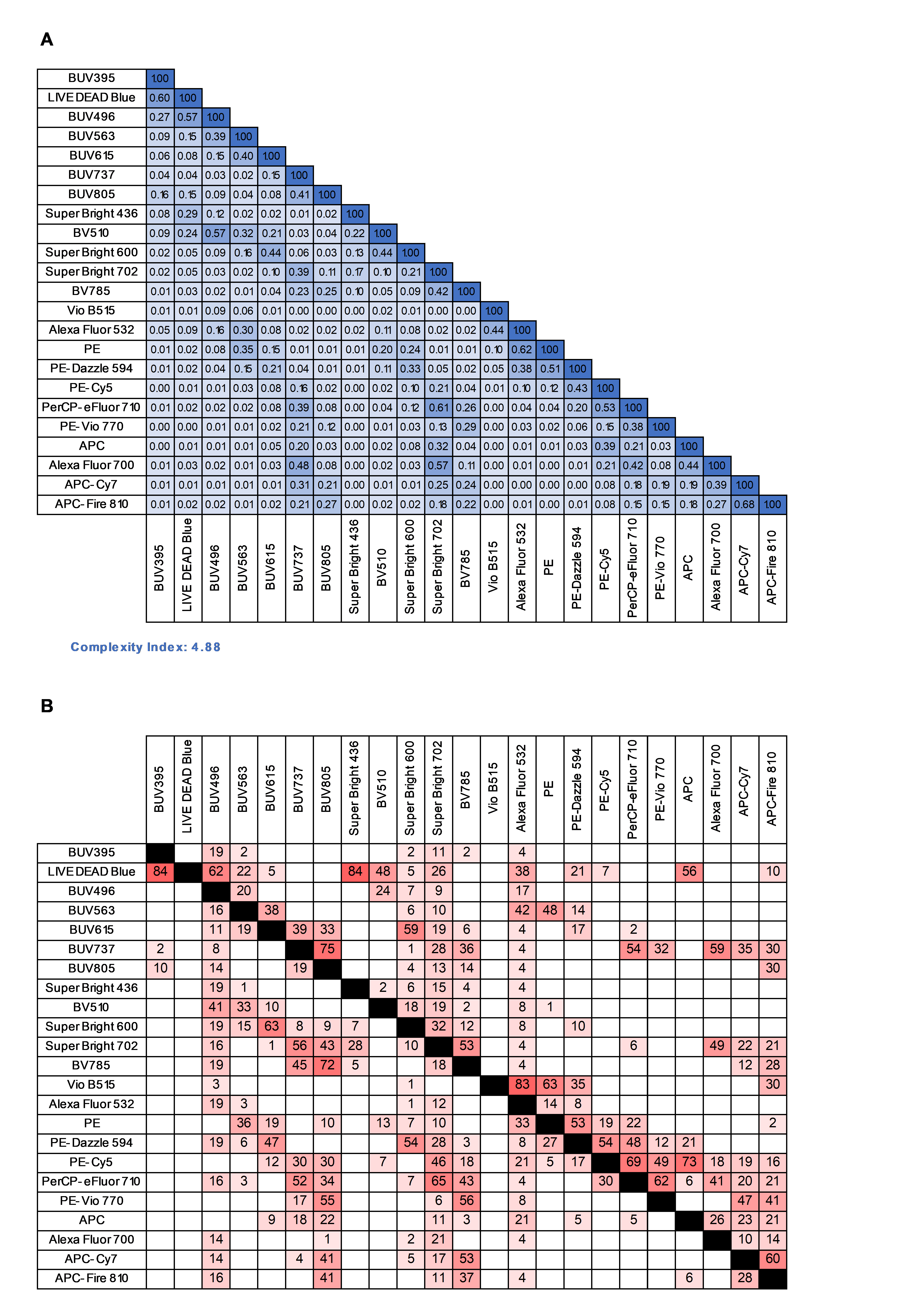

Supplement: Supporting Information 1 [file EMS207691-supplement-Supporting_Information_1.png]

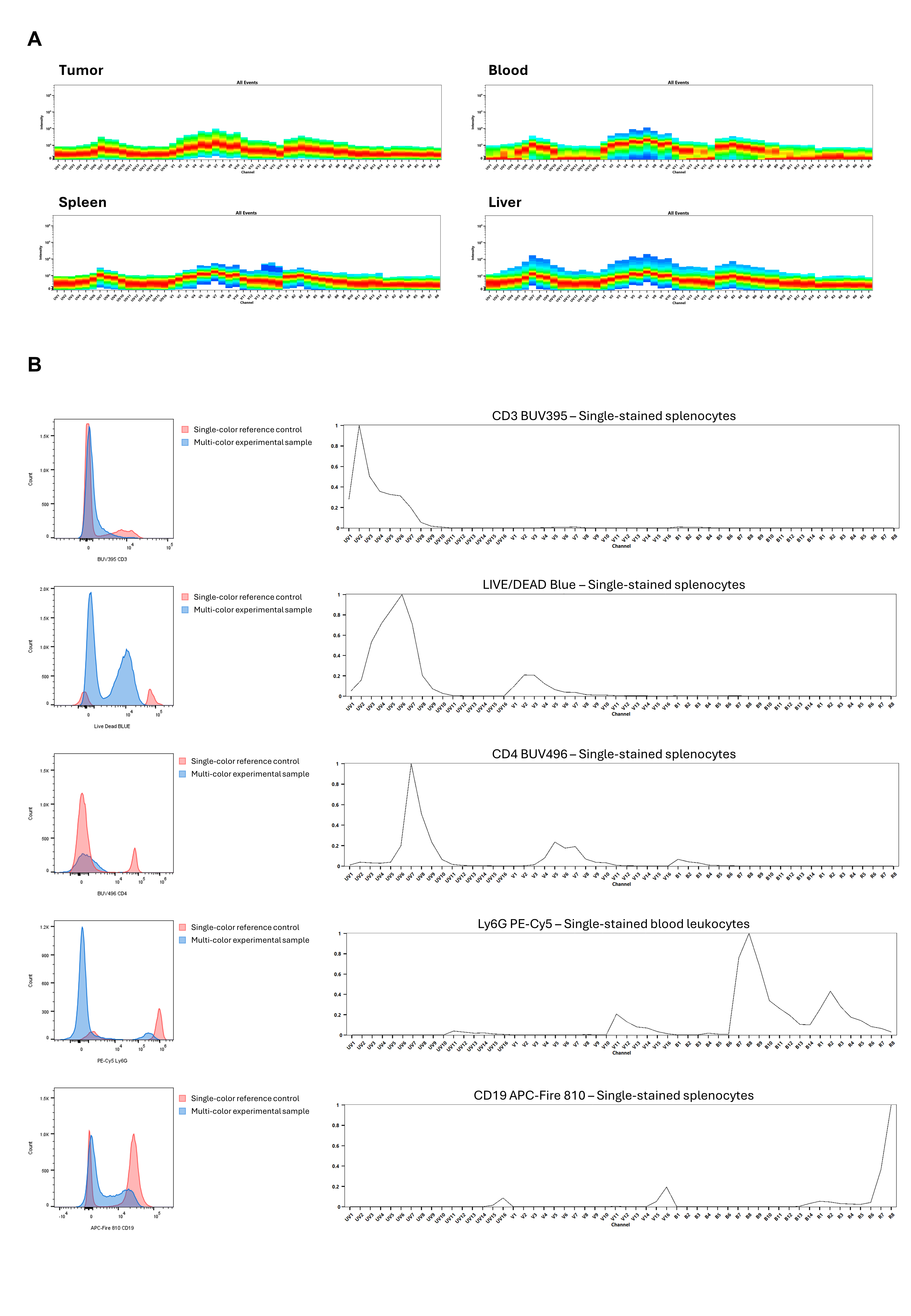

Supplement: Supporting Information 3 [file EMS207691-supplement-Supporting_Information_3.png]
