# Supplementary material for: Using spectral flow cytometry to characterize anti-tumor immunity in mouse models of cancer
Source: Curr Protoc. Author manuscript; Available in PMC 2025 Aug 12. (PMC7617995; doi:10.1002/cpz1.70032)
Supplement: Supporting Information 2 [file EMS207691-supplement-Supporting_Information_2.pdf]

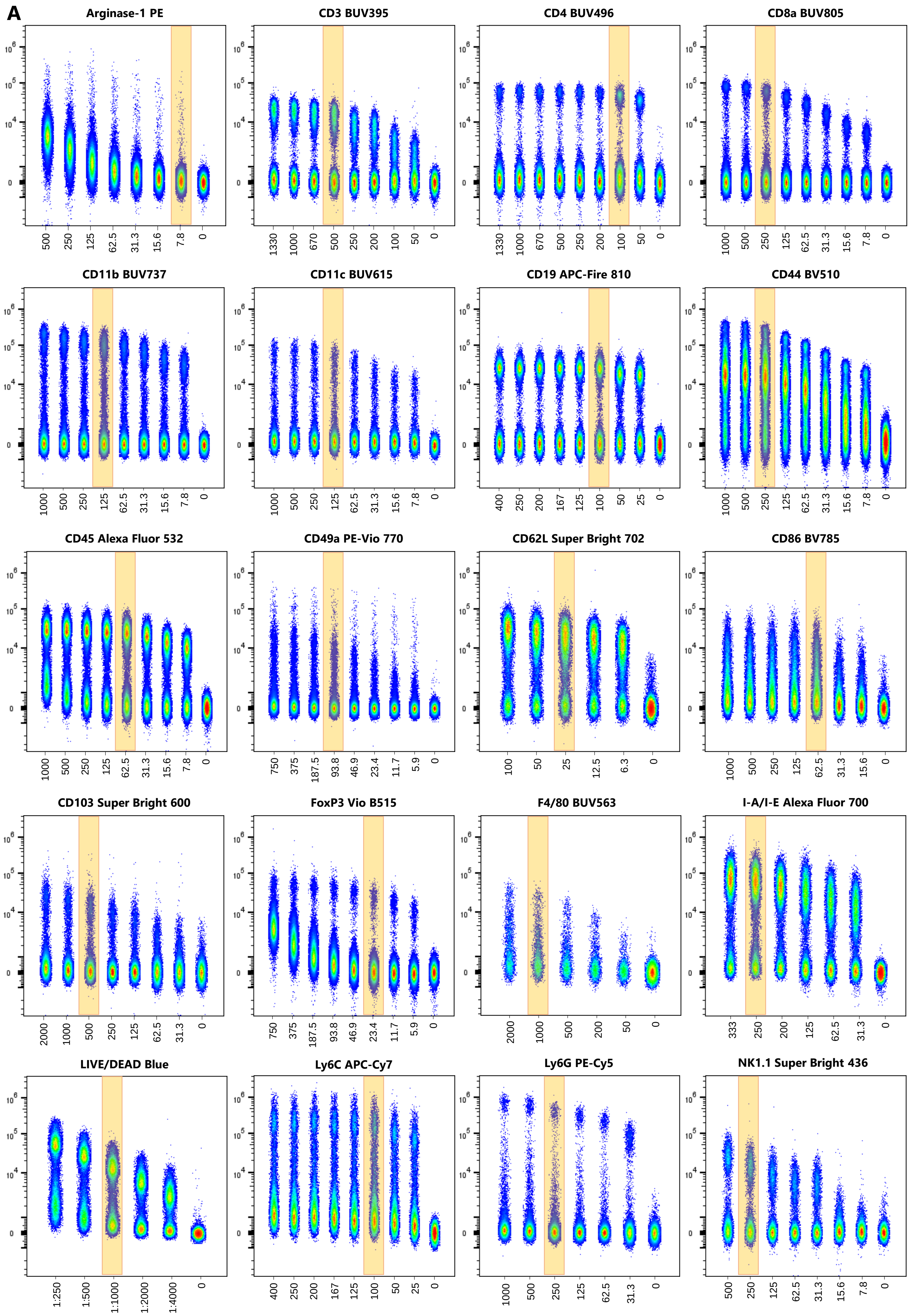
A
Arginase-1 PE
500 250 125 62.5 31.3 15.6 7.8 0
CD3 BUV395
1330 1000 670 500 250 200 100 50 0
CD4 BUV496
1330 1000 670 500 250 200 100 50 0
CD8a BUV805
1000 500 250 125 62.5 31.3 15.6 7.8 0
CD11b BUV737
1000 500 250 125 62.5 31.3 15.6 7.8 0
CD11c BUV615
1000 500 250 125 62.5 31.3 15.6 7.8 0
CD19 APC-Fire 810
400 250 200 167 125 100 50 25 0
CD44 BV510
1000 500 250 125 62.5 31.3 15.6 7.8 0
CD45 Alexa Fluor 532
1000 500 250 125 62.5 31.3 15.6 7.8 0
CD49a PE-Vio 770
750 375 187.5 93.8 46.9 23.4 11.7 5.9 0
CD62L Super Bright 702
100 50 25 12.5 6.3 0
CD86 BV785
1000 500 250 125 62.5 31.3 15.6 0
CD103 Super Bright 600
2000 1000 500 250 125 62.5 31.3 0
FoxP3 Vio B515
750 375 187.5 93.8 46.9 23.4 11.7 5.9 0
F4/80 BUV563
2000 1000 500 200 50 0
I-A/I-E Alexa Fluor 700
333 250 200 125 62.5 31.3 0
LIVE/DEAD Blue
1:250 1:500 1:1000 1:2000 1:4000 0
Ly6C APC-Cy7
400 250 200 167 125 100 50 25 0
Ly6G PE-Cy5
1000 500 250 125 62.5 31.3 0
NK1.1 Super Bright 436
500 250 125 62.5 31.3 15.6 7.8 0

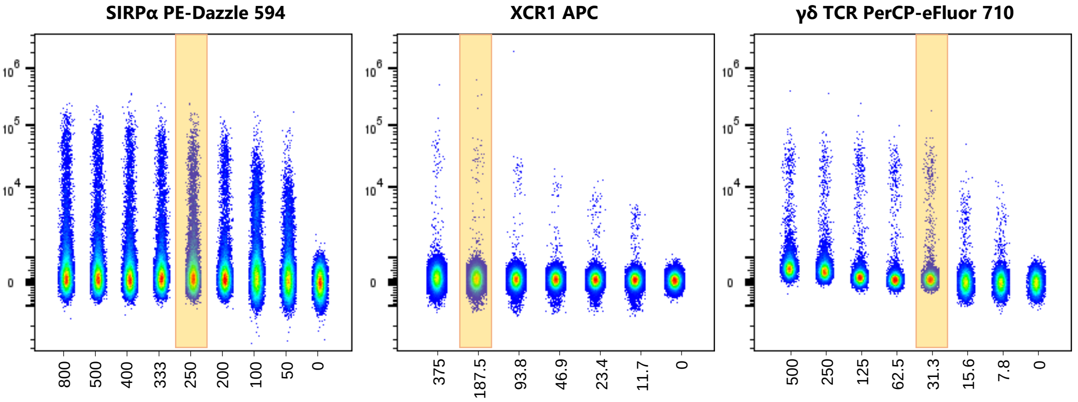

B

Reagent	Arginase-1 PE						
Concentration tested (ng/test)	500	250	125	62.5	31.3	15.6	7.8
Stain index	9.6	7.2	6.1	4.8	4.2	4.9	6.3
Relative stain index (%)	100.0	75.5	63.5	49.7	44.1	51.4	66.3
Reason for selected concentration	Higher titers cause smearing of the negative population						

Reagent	CD4 BUV496							
Concentration tested (ng/test)	1330	1000	670	500	250	200	100	50
Stain index	64.1	65.9	65.9	65.5	65.1	60.9	48.1	35.8
Relative stain index (%)	97.3	100.0	100.0	99.4	98.8	92.4	73.0	54.3
Reason for selected concentration	Comparable resolution to concentration with highest stain index							

Reagent	CD11b BUV737							
Concentration tested (ng/test)	1000	500	250	125	62.5	31.3	15.6	7.8
Stain index	196	181	152	134	117	86.8	57.1	41.8
Relative stain index (%)	100.0	92.3	77.6	68.4	59.7	44.3	29.1	21.3
Reason for selected concentration	Comparable results with 1/8 reagent							

Reagent	CD19 APC-Fire 810							
Concentration tested (ng/test)	400	250	200	167	125	100	50	25
Stain index	31.7	30.8	30.7	30.6	30.2	30.5	21.1	19.6
Relative stain index (%)	100.0	97.2	96.8	96.5	95.3	96.2	66.6	61.8
Reason for selected concentration	Nearly identical performance with 1/4 reagent							

Reagent	CD45 Alexa Fluor 532							
Concentration tested (ng/test)	1000	500	250	125	62.5	31.3	15.6	7.8
Stain index	12.9	19.7	24.6	26.3	26.1	22.4	16.3	12.1
Relative stain index (%)	49.0	74.9	93.5	100.0	99.2	85.2	62.0	46.0
Reason for selected concentration	Identical resolution compared to concentration with highest stain index							

Reagent	CD62L Super Bright 702				
Concentration tested (ng/test)	100	50	25.0	12.5	6.3
Stain index	27.5	21.1	17	15.1	11.6
Relative stain index (%)	100.0	76.7	61.8	54.9	42.2
Reason for selected concentration	Comparable results attained with lower titer				

Reagent	CD103 Super Bright 600						
Concentration tested (ng/test)	2000	1000	500	250	125	62.5	31.3
Stain index	23.5	21.4	18.5	12.9	9.4	3.6	2.6
Relative stain index (%)	100.0	91.1	78.7	54.9	40.0	15.3	11.2
Reason for selected concentration	Comparable results attained with lower titer						

Reagent	F4/80 BUV563				
Concentration tested (ng/test)	2000	1000	500	200	50
Stain index	6.23	6.81	5.68	5.36	2.68
Relative stain index (%)	91.5	100.0	83.4	78.7	39.4
Reason for selected concentration	Provides the highest stain index				

Reagent	LIVE/DEAD Blue				
Concentration tested (ng/test)	250	500	1000	2000	4000
Stain index	18	17.2	16.4	10.9	7.12
Relative stain index (%)	100.0	95.6	91.1	60.6	39.6
Reason for selected concentration	Higher titers cause shift in neg. population				

Reagent	Ly6G PE-Cy5					
Concentration tested (ng/test)	1000	500	250	125	62.5	31.3
Stain index	1045	996	716	585	418	113
Relative stain index (%)	100.0	95.3	68.5	56.0	40.0	10.8
Reason for selected concentration	Comparable results attained with lower titer					

Reagent	SIRPα PE-Dazzle 594							
Concentration tested (ng/test)	800	500	400	333	250	200	100	50
Stain index	26.4	25.6	26.6	26.2	24.4	22.6	6.2	4.45
Relative stain index (%)	99.2	96.2	100.0	98.5	91.7	85.0	23.3	16.7
Reason for selected concentration	Identical resolution compared to concentration with highest stain index							

Reagent	γδ TCR PerCP-eFluor 710						
Concentration tested (ng/test)	500	250	125	62.5	31.3	15.6	7.8
Stain index	45.5	38.8	59.9	53.5	49.9	7	4.2
Relative stain index (%)	76.0	64.8	100	89.3	83.3	11.7	7.0
Reason for selected concentration	Comparable results attained with lower titer						

Reagent	CD3 BUV395							
Concentration tested (ng/test)	1330	1000	670	500	250	200	100	50
Stain index	25.3	23.8	20.6	17.9	10.5	8.9	3.9	2.6
Relative stain index (%)	100.0	94.1	81.4	70.8	41.5	35.1	15.2	10.1
Reason for selected concentration	Identical resolution compared to concentration with highest stain index							

Reagent	CD8a BUV805							
Concentration tested (ng/test)	1000	500	250	125	62.5	31.3	15.6	7.8
Stain index	106	99.4	79.7	56.3	36.5	21.3	11.2	7.6
Relative stain index (%)	100.0	93.8	75.2	53.1	34.4	20.1	10.6	7.2
Reason for selected concentration	Comparable results with 1/4 reagent							

Reagent	CD11c BUV615							
Concentration tested (ng/test)	1000	500	250	125	62.5	31.3	15.6	7.8
Stain index	22.3	24.1	27.3	24.2	18.3	14.8	10.8	10.4
Relative stain index (%)	81.7	88.3	100.0	88.6	67.0	54.2	39.6	38.1
Reason for selected concentration	Identical resolution compared to concentration with highest stain index							

Reagent	CD44 BV510							
Concentration tested (ng/test)	1000	500	250	125	62.5	31.3	15.6	7.8
Stain index	8.4	8.1	7.3	5.1	3.3	2.3	2.6	3.3
Relative stain index (%)	100.0	97.3	87.5	60.3	39.8	27.6	30.7	39.9
Reason for selected concentration	Lower concentrations cause loss of distinction between $CD44^{-}$ / $CD44^{lo}$							

Reagent	CD49a PE-Vio770							
Concentration tested (ng/test)	750	375	187.5	93.8	46.9	23.4	11.7	5.9
Stain index	7.17	9.27	10.3	11.2	9.4	6.9	5.2	4.7
Relative stain index (%)	64.0	82.8	92.0	100.0	84.2	61.2	46.3	42.0
Reason for selected concentration	Provides the highest stain index							

Reagent	CD86 BV785						
Concentration tested (ng/test)	1000	500	250	125	62.5	31.3	15.6
Stain index	5.2	5.3	5.3	5.5	5.3	4.8	4.8
Relative stain index (%)	96.1	97.8	97.8	100.0	96.5	87.5	88.6
Reason for selected concentration	Comparable results attained with lower titer						

Reagent	FoxP3 Vio B515							
Concentration tested (ng/test)	750	375	187.5	93.8	46.9	23.4	11.7	5.9
Stain index	11.5	18.6	27.4	35.8	36.5	31.3	22.3	14.4
Relative stain index (%)	31.5	51.0	75.1	98.1	100.0	85.8	61.1	39.5
Reason for selected concentration	Comparable results attained with lower titer							

Reagent	I-A/I-E Alexa Fluor 700					
Concentration tested (ng/test)	333	250	200	125	62.5	31.3
Stain index	52.8	47.6	39.2	33	22.5	15.8
Relative stain index (%)	100.0	90.2	74.2	62.5	42.6	29.9
Reason for selected concentration	Comparable results attained with lower titer					

Reagent	Ly6C APC-Cy7							
Concentration tested (ng/test)	400	250	200	166.7	125	100	50	25
Stain index	116	123	122	118	120	118	79.7	61.3
Relative stain index (%)	94.3	100.0	99.2	95.9	97.6	95.9	64.8	49.8
Reason for selected concentration	Identical resolution compared to concentration with highest stain index							

Reagent	NK1.1 Super Bright 436						
Concentration tested (ng/test)	500	250	125	62.5	31.3	15.6	7.8
Stain index	28.5	19.8	13.7	9.4	9.1	3.3	2.5
Relative stain index (%)	100.0	69.5	48.1	32.8	31.9	11.7	8.7
Reason for selected concentration	Lower titer provides sufficient separation						

Reagent	XCR1 APC					
Concentration tested (ng/test)	375	187.5	93.8	46.9	23.4	11.7
Stain index	25.6	17.4	13.4	7.04	4.3	3.5
Relative stain index (%)	100.0	68	52.3	27.5	16.7	13.8
Reason for selected concentration	Comparable results attained with lower titer					
