# Supplementary material for: Using spectral flow cytometry to characterize anti-tumor immunity in mouse models of cancer
Source: Curr Protoc. Author manuscript; Available in PMC 2025 Aug 12. (PMC7617995; doi:10.1002/cpz1.70032)
Supplement: Supporting Information [file EMS207691-supplement-Supporting_Information.docx]

**SUPPORTING INFORMATION** *(optional):*

**Supporting Information 1.png**

Metrics for panel assessment – Similarity Index, Complexity Index, and Stain Index Reduction Matrix.

1. Similarity Index (SI) and overall Complexity Index (CI) for the fluorochromes in the panel. Pairwise comparison of all dyes included in the panel identify 0.68 as the highest SI in the panel, with a resultant overall CI of 4.88.
2. Stain Index Reduction (SIR) Matrix, also known as Cross-Stain Index Matrix, for the panel.

**Supporting Information 2.pdf**

Titration of all reagents in the definitive panel.

All reagents used in the definitive panel are shown in alphanumerical order. All titrations were performed on live singlets after splenocytes from wild type C57BL/6J mice were Fc-blocked, fixed and permeabilized, unless stated otherwise.

1. A concatenated dot plot for the visual assessment of the positive and negative populations at different concentrations is shown for each reagent. Y axis: fluorescence intensity; X axis: nanograms of reagents used to stain 10^6^ cells in 100 µL of staining buffer; yellow highlight: selected concentration. For LIVE/DEAD Blue, the dilution factor is shown, instead of the amount of reagent used.
2. For each reagent, the stain index for each tested concentration is shown, alongside the relative stain index (i.e., the ratio of stain index to maximal stain index, expressed as a percentage). When a different concentration was chosen to the one resulting in the highest stain index, the reason is clarified.

NK1.1 Super Bright 436 and γδ TCR PerCP-eFluor710 were titrated using live, Fc-blocked, fixed and permeabilized liver mononuclear cells (low-density cell fraction) from wild type C57BL/6J mice. F4/80 BUV563 and CD11c BUV615 were titrated after pre-gating on CD11b^+^ cells and I-A/I-E^+^ cells, respectively.

**Supporting Information 3.png**

Reference controls

1. Site-specific autofluorescence signal from unstained cells used for autofluorescence extraction during unmixing. “Tumor” and “Liver” refer to the low-density cell fraction of digested tumors and livers (largely corresponding to mononuclear cells), as explained in the main text. “Blood” refers to RBC-lysed whole blood (i.e., blood leukocytes). “Spleen” refers to splenocytes.
2. Single-color reference controls for which cells were used instead of beads. Left: Overlaid histograms showing the peak fluorescence signals for the positive and negative populations in the single-stained reference controls and multi-color experimental samples. Right: Corresponding full-spectrum fluorescent signal.
